# Solution structure of the HOIL-1L NZF domain reveals a conformational switch regulating linear ubiquitin affinity

**DOI:** 10.1016/j.jbc.2023.105165

**Published:** 2023-08-16

**Authors:** Erik Walinda, Kenji Sugase, Naoki Ishii, Masahiro Shirakawa, Kazuhiro Iwai, Daichi Morimoto

**Affiliations:** 1 Department of Molecular and Cellular Physiology, Graduate School of Medicine, Kyoto University, Kyoto, Japan; 2 Department of Molecular Engineering, Graduate School of Engineering, Kyoto University, Kyoto, Japan; 3 Division of Applied Life Sciences, Graduate School of Agriculture, Kyoto University, Kyoto, Japan

**Keywords:** ubiquitin, polyubiquitin, linear ubiquitin, LUBAC, HOIL-1L, protein–protein interactions, conformational dynamics

## Abstract

Attachment of polyubiquitin (poly-Ub) chains to proteins is a major posttranslational modification in eukaryotes. Linear ubiquitin chain assembly complex, consisting of HOIP (HOIL-1-interacting protein), HOIL-1L (heme-oxidized IRP2 Ub ligase 1), and SHARPIN (Shank-associated RH domain–interacting protein), specifically synthesizes “head-to-tail” poly-Ub chains, which are linked *via* the N-terminal methionine α-amino and C-terminal carboxylate of adjacent Ub units and are thus commonly called “linear” poly-Ub chains. Linear ubiquitin chain assembly complex–assembled linear poly-Ub chains play key roles in immune signaling and suppression of cell death and have been associated with immune diseases and cancer; HOIL-1L is one of the proteins known to selectively bind linear poly-Ub *via* its Npl4 zinc finger (NZF) domain. Although the structure of the bound form of the HOIL-1L NZF domain with linear di-Ub is known, several aspects of the recognition specificity remain unexplained. Here, we show using NMR and orthogonal biophysical methods, how the NZF domain evolves from a free to the specific linear di-Ub-bound state while rejecting other potential Ub species after weak initial binding. The solution structure of the free NZF domain revealed changes in conformational stability upon linear Ub binding, and interactions between the NZF core and tail revealed conserved electrostatic contacts, which were sensitive to charge modulation at a reported phosphorylation site: threonine-207. Phosphomimetic mutations reduced linear Ub affinity by weakening the integrity of the linear di-Ub–bound conformation. The described molecular determinants of linear di-Ub binding provide insight into the dynamic aspects of the Ub code and the NZF domain’s role in full-length HOIL-1L.

Protein ubiquitylation is one of the major post-translational modifications that occur in eukaryotic cells (1, 2). In contrast to other major modifiers such as phosphorylation and acetylation, ubiquitin (Ub) can be attached to target proteins not only as a single entity (monoubiquitylation) but also in the form of polymeric chains (polyubiquitylation) through concerted enzymatic action of a Ub-activating enzyme (E1), Ub-conjugating enzyme (E2), and a substrate protein-specific Ub ligase (E3). In these poly-Ub chains, subsequent Ub units are linked *via* the carboxyterminal glycine (G76) carboxylic acid group and the ε-amino group of one of seven internal lysine residues in Ub (K6, K11, K27, K29, K33, K48, and K63). Although initial focus was on K48-linked poly-Ub chains and their role in shuttling target proteins to the proteasome for degradation, as time progressed, nonproteolytic roles of Ub chains increasingly emerged over time. Intriguingly, an unexpected eighth modification was discovered in 2006, in which Ub linked *via* the α-amino group of its N-terminal methionine residue (M1) instead (3). This “linear” (M1-linked) chain is assembled exclusively by a unique Ub ligase enzyme complex called LUBAC (linear Ub chain assembly complex), which consists of the catalytic RBR-type E3 Ub ligase HOIP (HOIL-1-interacting protein), a second long-elusive RBR E3 ligase HOIL-1L (heme-oxidized IRP2 Ub ligase 1; which has recently been shown to have a regulatory [*i.e.*, attenuating] influence (4) on the catalytic activity of HOIP), and the accessory protein SHARPIN (Shank-associated RH domain–interacting protein) (5, 6, 7, 8). Molecular interactions of specific domains of HOIP, HOIL-1L, and SHARPIN with other intracellular proteins, as well as trimming of linear Ub chains by deubiquitinating enzymes OTULIN and CYLD, are an extremely active area of research (9, 10).

The major physiological functions of LUBAC-assembled linear Ub chains lie in mediating inflammatory responses, and a well-studied example is activation of the key transcription factor NF-κB, an immunological “master switch” playing a central role in mediating cell survival, proliferation, and inflammation (11). The specific signaling steps of LUBAC-mediated NF-κB activation have been extensively reviewed in the literature (11, 12). In line with the crucial involvement of LUBAC in both immune signaling and suppression of cell death, LUBAC components have been implicated in immune-related (autoinflammatory and autoimmune), cancer, and infectious diseases. Intriguingly, LUBAC component HOIL-1L has also been identified as an important factor in certain neuromuscular diseases (*e.g.*, in cardiomyopathy with polyglucosan bodies); however, its connection to immunological pathways and LUBAC function remains elusive (11, 13).

Apart from the UBAN domains of NEMO (12, 14) and the structurally close UBAN domains in ABIN and optineurin (UBAN refers to “ubiquitin binding in ABIN and NEMO”) (12), other proteins selectively binding to linear Ub chains, that is, having particularly high affinity for them, have been discovered. For example, A20, one of the target genes of NF-κB, harbors a domain termed ZF7 (*i.e.*, the seventh zinc finger domain) that has been shown to bind linear Ub chains avidly and that this interaction is important for suppression of NF-κB (15). Intriguingly, LUBAC component HOIL-1L *itself* has been shown to selectively bind to linear Ub chains *via* its NZF (Npl4 zinc finger) domain. This NZF domain contains a unique C-terminal region that is absent in other NZF domains—hereafter referred to as “NZF tail.” Both the core of the NZF domain and this tail bind to linear Ub, and it has been shown that disruption of this interaction attenuates NF-κB induction (16). Sato *et al.* (16) have provided a detailed structural basis for understanding the interaction between HOIL-1L NZF and linear poly Ub by X-ray crystallography. Specifically, they deciphered the interactions between the NZF core domain with both residues on a distal Ub and a proximal Ub subunit, as well as additional interactions between the NZF tail and proximal Ub. In the linear (head-to-tail) di-Ub molecule, the distal subunit refers to the N-terminal Ub moiety (harboring the free N-terminal amino group), whereas the proximal subunit is the C-terminal Ub moiety (harboring the free C-terminal carboxylic acid group). The most striking feature of the NZF–linear di-Ub interaction is its asymmetry, that is, the interaction sites on proximal and distal Ubs are different hydrophobic patches, which are bound by distinct residues of the NZF domain; in other words, the NZF domain needs to distinguish two virtually equivalent Ub moieties in solution. Although that crystal structure seemingly explains all aspects of the specificity of HOIL-1L for binding to linear Ub chains, several aspects of it remain unexplained. First, only the structure of the bound form of the NZF domain is known, yet the structure of the free form remains unknown—and as a result, any conformational changes occurring during binding have not been clarified. Second, the striking preference for M1-linked poly-Ub over the apparently similar (as first remarked by Komander *et al.* in 2009 (17)) K63-linked chain and the similarly binding site–compatible mono-Ub (which is available in much higher concentrations in cells (18)) remains insufficiently explained. Third, the role of M1-linked poly-Ub binding in the context of full-length HOIL-1L (and thereby LUBAC) is still scarcely understood.

Here, we combined solution-state NMR spectroscopy, molecular dynamics (MD) simulations, isothermal titration calorimetry (ITC), and structural modeling to fill these gaps in understanding regarding HOIL-1L, which may also grant new insight in a broader context of poly-Ub recognition specificity, that is, the recognition dynamics of the Ub code (1, 19).

## Results

### Solution-state poly-Ub binding by the HOIL-1L NZF domain

**Figure 1:**
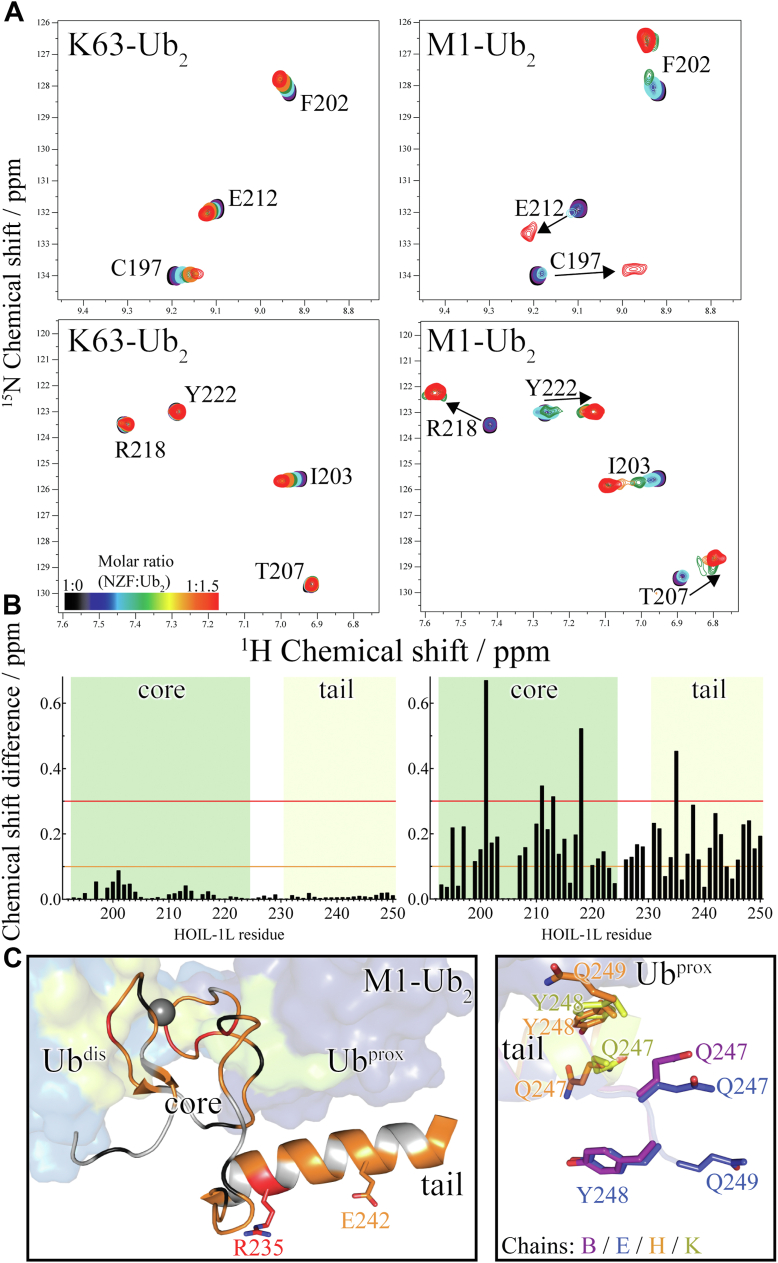
**Solution-state characterization of HOIL-1L NZF binding to ubiquitin (Ub) chains.***A*, *left*, ^1^H–^15^N heteronuclear single quantum coherence spectra showing the binding of (unlabeled) K63-linked di-Ub to the ^15^N-labeled HOIL-1L NZF domain. Several residues including Ile^203^ and Cys^197^ of the NZF core show distinct chemical shift perturbations in the fast exchange regime over the course of the titration. *Right*, binding of M1-linked di-Ub by the HOIL-1L NZF domain. Many residues of both the NZF core and NZF tail show slow-exchange and intermediate-exchange behavior over the course of the titration and overall, by comparison, much larger chemical shift changes are observed. *B*, averaged ^1^H–^15^N chemical shift differences between 0 and 1.5 eq. of added di-Ub as a function of HOIL-1L NZF residue (protein concentrations [in micromolar] of HOIL-1L NZF to [:] di-Ub are 100:0 [(0 eq.), 99.3:9.9 [0.1 eq.], 98.7:19.7 [0.2 eq.], 97.4:39.0 [0.4 eq.], 94.9:75.9 [0.8 eq.], 93.8:93.8 [1.0 eq.], 90.9:136.4 [1.5 eq.]). *Left*, titration of the NZF with K63-linked di-Ub. *Right*, titration of the NZF with M1-linked di-Ub. The core and tail regions of the HOIL-1L NZF domain are indicated by *green* and *yellow shading*, respectively. To enable comparison among different di-Ub types, the following criteria have been selected as thresholds: *orange line*, chemical shift difference = 0.1 ppm (parts per million); *red line*, chemical shift difference = 0.3 ppm. Although the interaction with M1-linked di-Ub is evident, several amino acids of the NZF core appear to weakly interact with K63-linked di-Ub. We note that the NZF domain contains six proline residues for which no titration information is available. *C*, *left*, visualization of specific binding of M1-linked di-Ub as observed by solution NMR mapped on the NZF/M1-linked di-Ub cocrystal structure (PDB ID: 3B08 (16)). The NZF domain is shown as *cartoon* and di-Ub as transparent surface representation (*gray sphere*, Zn^2+^ ion). Residues of the NZF are colored according to the observed chemical shift differences (as defined in ([*B*]). Although many chemical shift changes are directly explained by the crystal structure, sites remote from and even facing away from di-Ub were also found to show high chemical shift differences upon di-Ub binding as highlighted for Arg^235^ and Glu^242^. *Right*, structural heterogeneity near the carboxyterminal end of the NZF tail helix. Shown is an overlay of the four distinct chains of the NZF found in the asymmetric unit of PDB ID 3B08 (*cartoon* and side chain colored according to chain identifier as indicated). In this crystal structure, there appear to be several distinct conformations for the side chains of the three carboxyterminal residues (the last residue, Gln^250^ is not observed in the crystal structure, whereas Gln^249^ is only observed for two of the four chains and in quite different conformations). HOIL-1L, heme-oxidized IRP2 Ub ligase 1; NZF, Npl4 zinc finger; PDB, Protein Data Bank.

Based on surface plasmon resonance (SPR) experiments and crystallographic data, it has been established that the (mouse) HOIL-1L NZF domain including the NZF tail (hereafter referred to as “HOIL-1L NZF”) selectively binds to M1-linked poly-Ub chains (16). However, experimental evidence from solution scattering studies (20, 21) and computational studies (22, 23) indicates marked similarities between free, that is unbound, K63-, and M1-linked chains. Importantly, both chain types can form similarly open and extended (Fig. S1; Table S1) conformations in solution. This similarity arises from the close proximity of the K63 ε-amino group and the M1 α-amino group (17) and suggests that the conformational flexibility in the K63-linked chain might allow it to occasionally adopt “M1-like” conformations, which would allow it to interact with HOIL-1L NZF. Therefore, to reassess the poly-Ub binding specificity of HOIL-1L NZF in solution and in a site-specific manner, we prepared ^15^N-isotope-labeled purified HOIL-1L NZF protein samples and conducted NMR titration experiments. In titration experiments using K63-linked di-Ub, we observed weak binding to the HOIL-1L NZF domain (Fig. 1*A*, *left*). For example, residues Cys^197^, Phe^202^, Ile^203^, and Glu^212^ of the NZF core (Fig. 1*A*, *left*) showed clearly detectable chemical shift changes upon increasing the di-Ub to NZF molar ratio. Three of the residues shown in Figure 1*A*, *left* (Phe^202^, Ile^203^, and Glu^212^) have previously (16) been implicated in binding to *M1*-linked di-Ub on the distal Ub moiety, whereas Cys^197^ is a structural Zn^2+^-coordinating residue. Nevertheless, the overall binding affinity toward *K63*-linked di-Ub was deemed to be rather weak, as all observed chemical shift changes were clearly in the fast-exchange regime on the NMR timescale. Least-squares fitting of the chemical shift changes as a function of the concentrations yielded a comparably high dissociation constant (*K*_*d*_) of approximately 224 μM (Table S2). Thus, K63-linked di-Ub bound even more weakly than mono-Ub (*K*_*d*_ = 131 μM; Table S2) to the NZF domain despite identical molecular composition. Although this may seem paradoxical, a recent study reported found only 10% of K63-linked di-Ub in solution in an extended conformation, whereas the majority of conformers adopted one of various compact structures; such compact conformers might occlude binding site residues, making these residues not readily available for association with the NZF domain (24). Taken together, the NMR titration data are in line with the previously published SPR-based conclusion (16) that the NZF domain binds K63-linked di-Ub with very low affinity.

In stark contrast, titration experiments using M1-linked di-Ub indicated a highly specific and high-affinity binding process. NMR amide resonances either showed significant broadening over the course of the titration (indicating intermediate exchange regime on the NMR timescale) or started to disappear in the free state to reappear in the bound state (indicating slow exchange). For instance, the signal corresponding to Arg^218^ gradually disappeared in the free state and then reappeared in the bound state (Fig. 1*A*, *right*), whereas Phe^202^, Ile^203^, and Glu^212^ showed gradual chemical shift changes albeit with marked line broadening over the course of the titration. As these two residues map to the distal Ub-binding site in the reported cocrystal structure, their involvement in distal Ub binding explains their large chemical shift perturbation (CSP; Δδ) and line broadening (Δ*R*_2_) upon interaction with M1-linked di-Ub. Similarly, Thr^207^, a residue close to the proximal Ub-binding site, exhibited tremendous line broadening at intermediate molar ratios. For Thr^207^, interestingly at such molar ratios, multiple broad resonances are observable (Fig. 1*A*, *right*; *green spectrum*), indicating the presence of multiple intermediate states between the free and final bound states. The marked preference and high specificity in binding toward M1-linked di-Ub are also apparent in the side-chain indole amide resonance of Trp^195^, which albeit structurally buried is located close to the proximal Ub-binding site (Fig. S2). The full titration spectra representing all residues of the HOIL-1L NZF domain are shown in Fig. S3.

Taken together, K63-linked di-Ub appears to weakly interact with the NZF core, but not with the NZF tail (Fig. 1*B*), whereas M1-linked di-Ub specifically interacted with both regions. The NMR titration experiments are in fine agreement with the available crystallographic data, demonstrating specific binding toward M1-linked poly-Ub *via* both distal and proximal binding sites, whereas the K63-linked chain is only transiently bound by the NZF core and rapidly dissociates under fast-exchange kinetics (assuming the fast *k*_ex_ reflects a fast *k*_off_, with *k*_on_ being diffusion limited (25)).

### Structural implications from solution-state binding experiments

Interestingly, not all chemical shift changes observed in the titration experiment could be unequivocally explained by the cocrystal structure of the HOIL-1L NZF/M1-linked di-Ub complex, in other words, by the structure of the *bound state*, alone. When mapping the chemical shift changes observed in the NMR titration onto this crystal structure, indeed most interacting residues mapped to Ub-binding sites; however, a couple of residues demanded further explanation. For instance, the side chain of Arg^235^ of the NZF tail faces away from the binding sites of both Ub moieties; yet Arg^235^ displays significant chemical shift changes upon binding to M1-linked di-Ub. Likewise, the NZF tail residue Glu^242^ side chain points away from the proximal Ub moiety; in addition, even more distal (*i.e.*, carboxyterminal) residues Glu^243^ and Arg^246^ of the NZF tail are not in contact with proximal Ub, whereas Gln^250^ is missing in the crystal structure and Gln^247^, Tyr^248^, and Gln^249^ are observed in multiple distinct conformers at different distances seen from proximal Ub (Fig. 1*C*); yet all these residues show significant chemical shift changes upon binding M1-linked di-Ub. In part, secondary chemical shift changes might be responsible for the CSP of these residues. Nevertheless, taken together, these observations suggested the possibility that the NZF domain undergoes some sort of an intriguing structural change upon binding that is not evident from the bound state cocrystal structure alone.

### Solution structure of the HOIL-1L NZF domain in the free form

**Figure 2:**
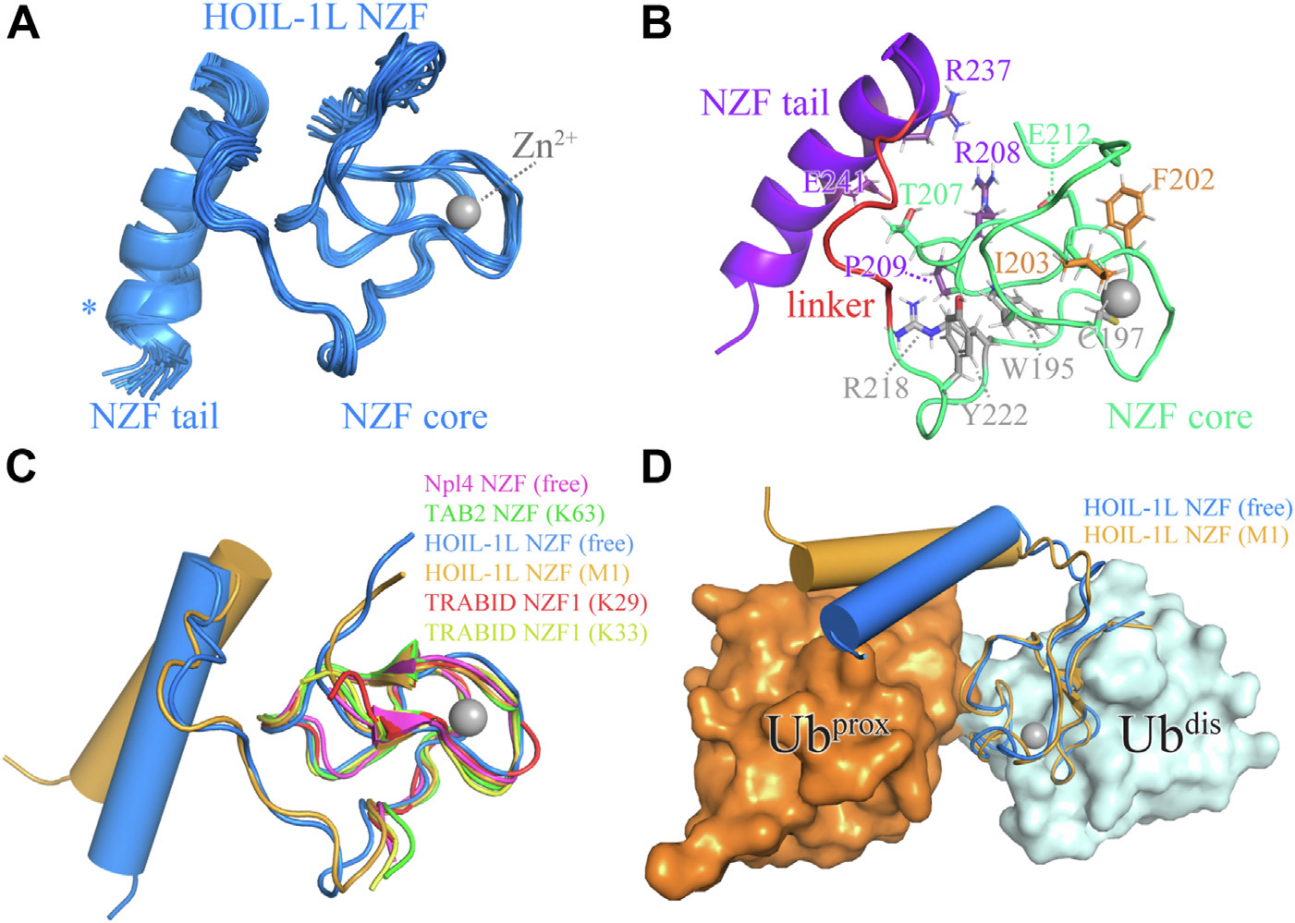
**Solution structure of the free HOIL-1L NZF domain.***A*, *blue cartoon*, ensemble of the 20 lowest energy conformers as obtained by CYANA (69) of the mouse HOIL-1L NZF domain in the absence of ubiquitin (Ub). *Gray sphere*, Zn^2+^ ion of the lowest energy conformer; Zn^2+^ ions of other conformers omitted for clarity. Details of the structure determination are given in Table S3. *Asterisk* (∗), see the Discussion section for additional considerations on the stability of the carboxyterminal end of the NZF tail helix. *B*, elements of the overall NZF architecture: core (*green*), tail (*purple*), and the linker between core and tail (*red*); precise residue boundaries for these elements are given in the supporting information. The depicted structure corresponds to the minimum-energy structure of the ensemble shown in (*A*) slightly rotated to visualize side chains. Several representative residues from proximal (*purple*) and distal (*orange*) Ub-binding sites are shown. *Gray-colored residues* show examples where chemical shift perturbation was described in the context of Figure 1 but are not directly part of a known Ub-binding surface; *green-colored residues* are Thr^207^ on the interface between NZF core and tail, and Glu^212^ is close to both the distal and proximal Ub moiety. *C*, comparative overlay of the HOIL-1L NZF solution structure (lowest-energy conformer; *blue*) with other published structures of proteins harboring NZF family domain members: Npl4 (free form; *magenta*), TAB2 (K63-linked di-Ub bound; *green*), TRABID1 (K29-linked di-Ub-bound form in *red*; K33-linked di-Ub-bound form in *yellow*), and the crystal structure of the HOIL-1L domain in the M1-linked di-Ub-bound form (*orange*; PDB IDs and references are given in the main text). All Ub units from these structures are omitted for clarity. *D*, comparative overlay of the HOIL-1L NZF solution structure (lowest-energy conformer; *blue cartoon*) with the crystal structure of the HOIL-1L domain (M1-linked di-Ub-bound form, *orange cartoon*) also showing the proximal (*orange surface*) and distal (*light blue surface*) Ub moieties in this structure. Transition from the free to the bound form of the NZF domain appears to entail rotation of the NZF tail outward away from the core and toward the proximal Ub moiety. For simplicity, cylindrical helix representations were chosen in (*C*) and (*D*); however, we note that this representation omits the small kink in the helix observed in (*A*) and the conformational dynamics of the helix described subsequently. HOIL-1L, heme-oxidized IRP2 Ub ligase 1; NZF, Npl4 zinc finger; PDB, Protein Data Bank.

To gain insight into possible structural changes in the HOIL-1L NZF domain occurring upon linear poly-Ub binding, we set out to elucidate the yet-missing solution structure of the NZF domain in the free form. The resulting structural ensemble as determined by using solution-state NMR spectroscopy based on NOE-derived distance and residual dipolar coupling (RDC)-derived orientational restraints (Tables S3 and S4) is shown in Figure 2*A*. Elements of the NZF domain architecture, that is, the NZF core, tail, and core–tail linker region, are defined in Figure 2*B*, and side chains from key regions of the structure are highlighted.

The structure of the NZF core zinc finger motif adopts a canonical fold, as its backbone fold is virtually identical to that of other NZF domains previously described in the literature (Fig. 2*C*). For example, the HOIL-1L NZF backbone overlays well with the TRABID NZF1 domain, which has been reported in two different cocrystal structures with, respectively, K29- and K33-linked di-Ub (Protein Data Bank [PDB] ID: 5AF6; PDB ID: 4S1Z (26, 27)), the TAB2 NZF domain (PDB ID: 2WX0 (28)), the Npl4 NZF domain (PDB ID: 1NJ3 (29)), as well as the HOIL-1L NZF domain itself in the cocrystal structure with M1-linked di-Ub, that is, the bound form (PDB ID: 3B08 (16)). The NZF domains of these proteins do not contain an NZF tail, which has only been identified for HOIL-1L and thus to date appears to be a unique feature of HOIL-1L.

Compared with the structure of the M1-linked di-Ub-bound form, however, the NZF tail adopts a notably changed conformation in the free form. In the structure of the free form, the NZF tail α-helix is tilted more markedly toward the NZF core (Fig. 2*D*) than in the di-Ub-bound form. This might hint at the necessity for a conformational change during poly-Ub binding, that is, an outward flip of the NZF tail to grasp the proximal Ub moiety. Since the proximal Ub moiety is known to interact with *both* the NZF tail (*via* tail residues Arg^237^ and Gln^241^; Fig. 2*B*, *purple*) *and* the NZF core (*via* residues Arg^208^ and Pro^209^; Fig. 2*B*, *purple*) (16), one simple hypothetical mechanism might be that the NZF core engages to make several initial contacts to proximal Ub upon encounter, which triggers an outward rotation of the NZF tail residues to “lock-in” proximal Ub in a fully bound conformation; alternatively, the tail might engage first, after which the NZF core rotates toward its binding site on proximal Ub; possibly both mechanisms occur independently in solution. Such a relative rotation between core and tail could be facilitated by the presence of the hinge sequence (Ile^224^–Pro^230^; Fig. 2*B*, *red*), which is devoid of recognizable secondary structure elements in both the free and bound forms and thus might possess a certain degree of backbone conformational flexibility. Indeed, the dihedral angles differed between the free and bound structures, with the largest change seen in the region Ser^227^–Gln^229^ (Table S5), consistent with the significant chemical shift changes observed for this part of the linker region (Fig. 1).

In addition to this difference in the overall orientation, the NZF tail displays increased curvature in the free form. The bound form features a straight and extended helix for all residues close to proximal Ub (residues Glu^232^–Gln^247^), whereas the solution structure of the free form reveals a kink near the carboxyterminal end of this helix (Fig. 2*A*). Although a kink causing the helical curvature is not unusual in general (30), this difference in conformation compared with the bound form indicates that proximal Ub binding might stabilize and thereby *straighten* out the NZF tail. In other words, the absence of a bound proximal Ub moiety in the free form might account for a large degree of conformational flexibility in the carboxyterminal part of the NZF tail helix (residues Gln^247^–Gln^250^) (31). Such changes in helix orientation and helix conformational flexibility upon binding also help to explain the observed CSPs for Arg^235^ and Glu^242^, which are in direct contact with neither proximal Ub nor distal Ub (Fig. 1). Taken together, although the NZF core structurally closely resembles the bound form, both the relative orientation and the flexibility of the NZF tail appear to differ between the free and bound states.

### Structural dynamics of the HOIL-1L NZF domain in solution

**Figure 3:**
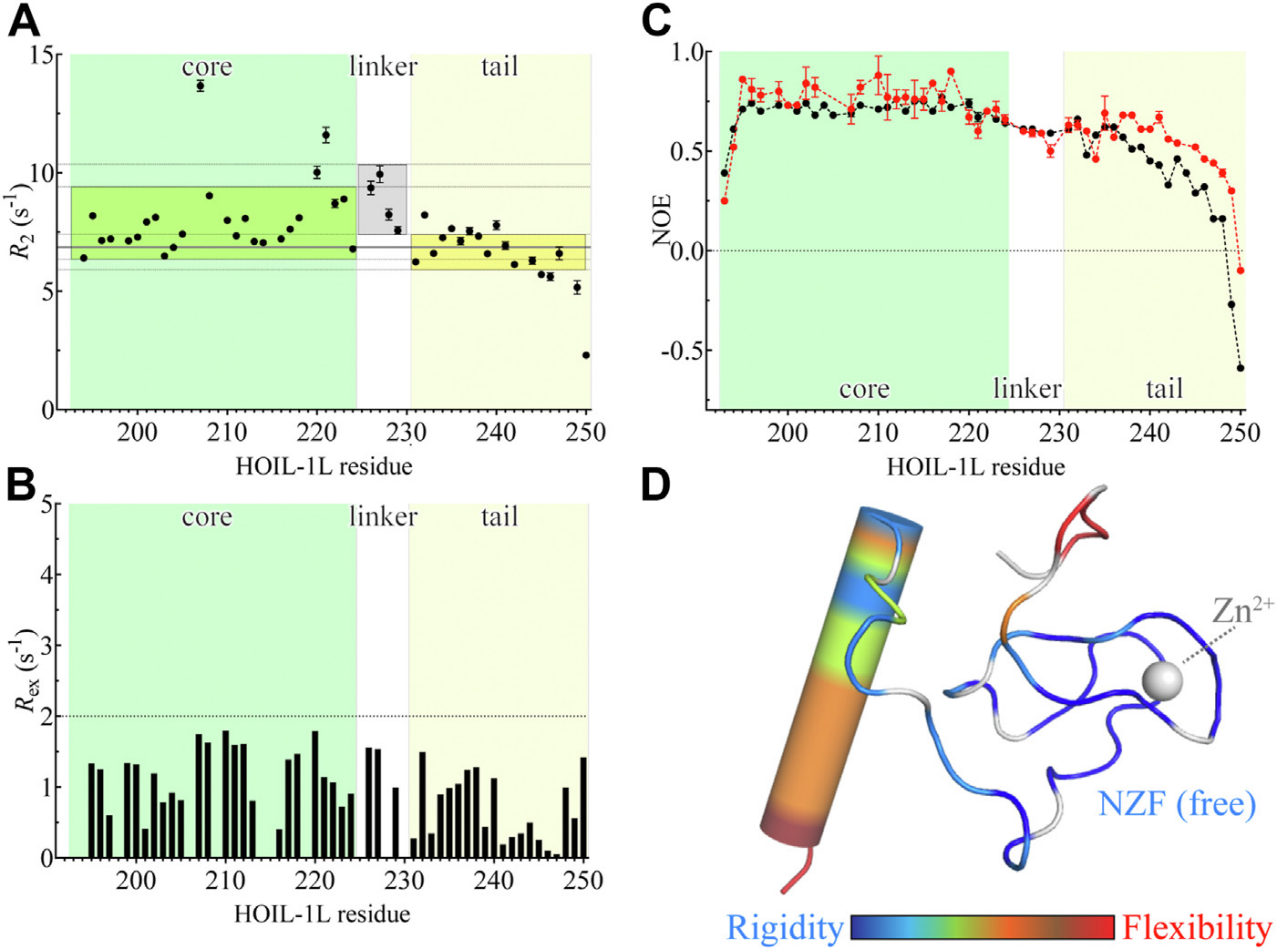
**Structural dynamics in the HOIL-1L NZF domain.***A*, markers show the measured ^15^N transverse relaxation rates (*R*_2_) of the free HOIL-1L NZF domain; error bars indicate the derived experimental uncertainty in the measured *R*_2_ values (from Monte–Carlo simulations with 100 iterations). *Gray line*, the theoretically expected *R*_2_ value for the entire NZF domain (7.8 s^−1^). *Green*-, *gray*-, and *yellow-shaded boxes* show the average ± 1 standard deviation of the measured *R*_2_ values for, respectively, the NZF core, the region linking the NZF core and tail regions, and the NZF tail. *Dashed gray lines* show the boundaries of the *shaded boxes*. *B*, the absence of micro-to-millisecond conformational dynamics in the isolated NZF domain at 298 K. *Bars* show the per-residue *R*_ex_ rates measured by ^15^N *R*_2_ relaxation dispersion, followed by fitting of the *R*_2_ relaxation dispersion profiles to the Carver–Richards equation. *Dashed line*, cutoff value (*R*_ex_ = 2 s^−1^; *i.e.*, no residue met this criterion for further analysis). *C*, ^15^N–{^1^H} hnNOE values for the free NZF domain (*black markers*) and the NZF domain in the presence of 2 molar equivalents of M1-linked diubiquitin (*red markers*). Data represent the average ± 1 standard deviation of three repeated measurements. *D*, visualization of the hnNOE data of the free NZF domain (*i.e.*, from *C*; *black markers*) on the structure of the free NZF domain. Backbone flexibility as judged from hnNOE measurements is colored from *dark blue* (highly rigid) to *red* (highly flexible) as indicated (proline residues are drawn *white*; at the *top*, *red-colored residues* indicate the plasmid-derived N-terminal GPLGSPE sequence). hnNOE, heteronuclear NOE; HOIL-1L, heme-oxidized IRP2 ubiquitin ligase 1; NZF, Npl4 zinc finger.

While these results indicated that the conformational flexibility of the NZF domain underwent changes during the interaction with (and thus appeared to play a role in binding to) linear Ub chains, the degree of conformational flexibility in the free NZF structure remained to be verified as it is not immediately evident from NMR structure determination alone. Therefore, to experimentally gauge the backbone conformational flexibility in the free NZF domain, we conducted ^15^N transverse spin relaxation rate measurements, a technique sensitive to nanosecond protein backbone motions. If two domains of unequal size in a multidomain protein undergo largely independent diffusion in solution, these domains experience different hydrodynamic friction so that different average *R*_2_ values are observed for these individual domains (32). Here, however, the measured transverse relaxation rates confirmed that the NZF domain predominantly diffuses as a single domain in solution overall (Fig. 3*A*): the average relaxation rates for the NZF core and tail regions were, 7.9 s^−1^ and 6.7 s^−1^, respectively. This is close to the theoretically expected (*i.e.*, calculated) in-phase ^15^N *R*_2_ value of 6.9 s^−1^ for the combined NZF (core–linker tail) domain (at 298 K and 16.4 T; calculated rotational correlation time of 3.8 ns; see later for the possible impact of exchange contributions, *R*_ex_, on the measured *R*_2_) (Fig. 3*A*; *gray line*). This overall uniformity in the measured transverse relaxation rates indicated that both the NZF core and the tail diffused together in solution, consistent with the determined NMR structure (Fig. 2*A*).

Because of the importance of both the NZF core and the tail in the function of the NZF domain, we also examined whether any micro-to-millisecond conformational exchange such as a hypothetical detachment of the tail from the core process took place in the isolated NZF domain. However, measurements of exchange contributions to the ^15^N transverse relaxation rate (*R*_ex_) by *R*_2_ relaxation dispersion experiments showed an *R*_ex_ <2 s^−1^ for all residues of the NZF domain, which is often used as a cutoff for *R*_2_ dispersion analysis. Thus, in the isolated NZF domain, no measurable micro-to-millisecond motion between the core and tail region appeared to take place, confirming the overall stable integrity of the NZF core–tail domain even in the absence of bound di-Ub.

To further narrow down the conformational behavior of the NZF domain in solution, we conducted ^15^N–{^1^H} heteronuclear NOE (hereafter: hnNOE) measurements. This experiment can distinguish very rapid (hnNOE <0.2) from medium (hnNOE ∼0.5) and slow (hnNOE >0.7) backbone motions and thus reports on main-chain conformational flexibility in proteins on the pico-to-nanosecond timescale with higher values indicating conformational rigidity. The NZF core showed hnNOE values of approximately 0.7 (Fig. 3*C*, *black*; Fig. 3*D*), confirming that the NZF core is a rather rigid stably folded entity in solution. Interestingly, the region linking the NZF core and tail region showed only slightly lower hnNOE values (Fig. 3*C*, *black*; Fig. 3*D*), suggesting that this linker region is not fully flexible in solution despite the lack of secondary structure elements in this region. In the NZF tail region, the hnNOE values showed a gradual decline toward the C terminus (Fig. 3*C*, *black*; Fig. 3*D*), reflecting a much higher degree of conformational flexibility in the C-terminal half of the NZF tail helix. This residual local flexibility might confer the NZF tail with one of the unique characteristics of intrinsically disordered proteins, which have been shown to efficiently bind to target ligand molecules by being able to rapidly sample a large number of conformations in solution (33).

To assess whether changes in the conformational flexibility of the NZF domain occur upon specific (M1) poly-Ub binding, we also conducted the hnNOE measurements in the presence of M1-linked di-Ub. Importantly, hnNOE values were markedly increased in the NZF tail (Fig. 3*C*; *red*). In other words, the presence of bound M1-linked di-Ub resulted in stabilization of the conformation of the NZF tail. Although far C-terminally located residues such as Gln^249^ and Gln^250^ still displayed rather low hnNOE values, this behavior is consistent with the crystal structure in which these residues retain conformational flexibility even in the M1-di-Ub-bound form (Fig. 1*C*). Moreover, structural stabilization because of M1-linked di-Ub binding was also detectable in the NZF core, albeit to a lower extent. Taken together, backbone flexibility in the NZF domain differs between the free and M1-linked di-Ub-bound forms, with conformational stabilization taking place upon binding. Conversely, the residual conformational flexibility in the NZF tail of the free form might be important in the early stages of the binding interaction.

### Internal noncovalent interactions between the NZF core and NZF tail

**Figure 4:**
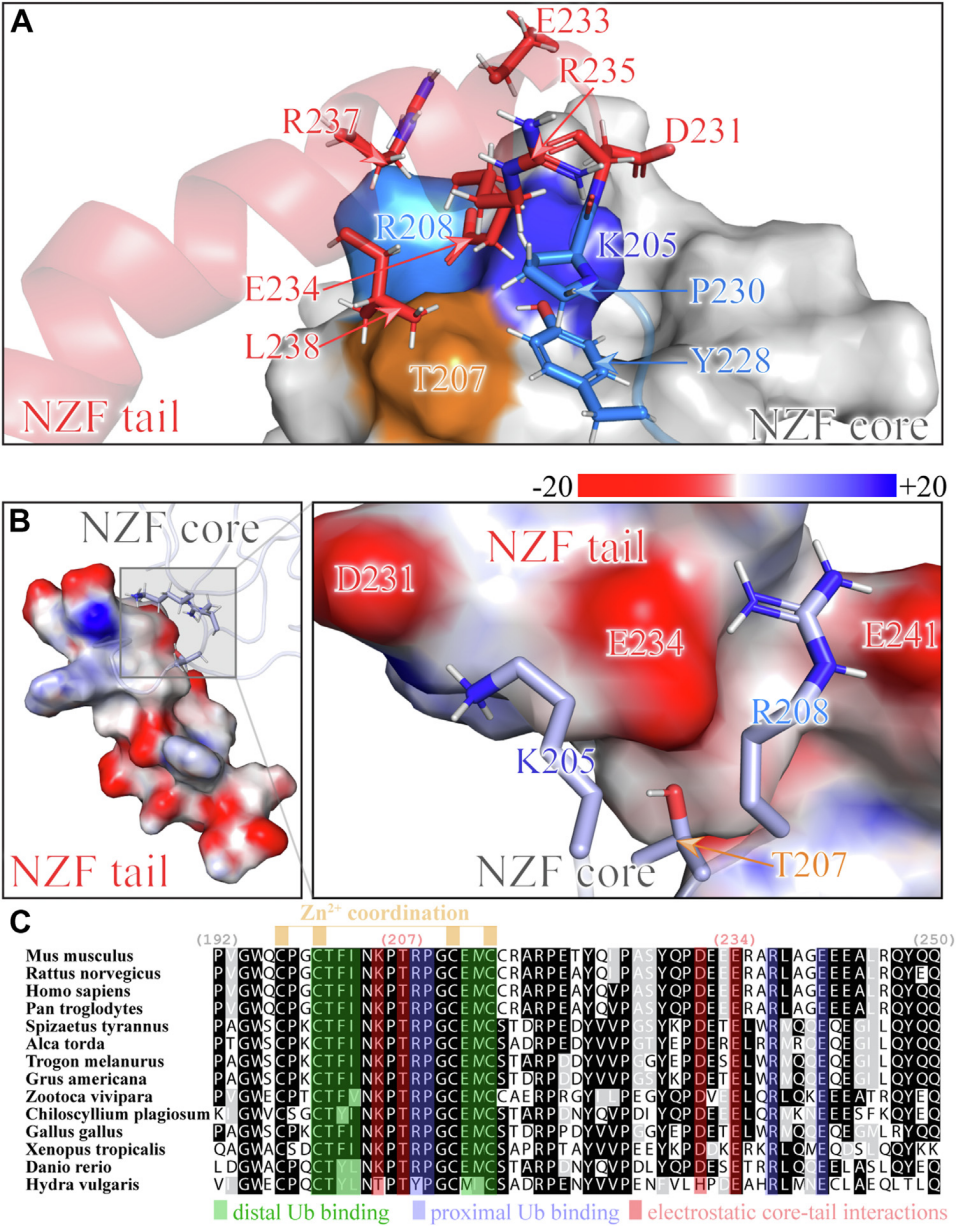
**Interactions between NZF core and tail regions in solution.***A*, close-up view highlighting interactions between the α-helix of the NZF tail (*red*; *ribbon representation*) and the NZF core (*gray*; *surface representation*). The region connecting the core and tail is also shown (*blue*; *ribbon representation*) with interacting side chains as *sticks*. Important NZF tail residue side chains are drawn as *red sticks*, whereas selected NZF core residues are colored in *blue* (Lys^205^), *cyan* (Arg^208^), and *beige* (Thr^207^). The proximity of negatively charged NZF tail side chains and positively charged NZF core residues is evident. *B*, visualization of the overall electrostatic potential of the NZF tail (surface representation colored according to charge) with positively charged NZF core side chains interacting with this surface. *Left*, overall view of the NZF tail; *right*, zoom-up view. Three critical points of negative charge density on the NZF tail are found on Asp^231^, Glu^234^, and Glu^241^. Positively charged NZF core side chains Lys^205^ and Arg^208^ snugly fit into this surface of negative charge density. Although Thr^207^ is polar but uncharged, it directly faces a point of high negative charge density in this structure. Electrostatic potential as derived by Adaptive Poisson–Boltzmann Solver (APBS) calculations (35) is given at the top in units of *k*_b_*T*/*e* (*i.e.*, 1 unit corresponds to 25.85 mV at 300 K). *C*, sequence homology analysis of the HOIL-1L NZF as identified by BLAST (70), aligned by Clustal Omega (71), and shaded by *BoxShade* (identical residues: *black*; similar residues: *gray*). In addition, residues known to be part of the distal (*green shading*) and proximal (*indigo shading*) ubiquitin-binding sites are highlighted. Conserved cysteine residues involved in Zn^2+^ coordination are shown at the *top* in *beige*. Residues involved in electrostatic core–tail interactions are highlighted by *red shading*. Residue numbers corresponding to mouse HOIL-1L are given at the *top*. HOIL-1L, heme-oxidized IRP2 ubiquitin ligase 1; NZF, Npl4 zinc finger.

Next, we inspected the structural interface between the NZF core and NZF tail helix to understand their relative relationship in the structure of the free HOIL-1L NZF domain. As shown in Figure 4*A*, multiple side chains of the NZF tail helix interacted with the surface of the NZF core. For example, the aliphatic side chain of Leu^238^ was found in close vicinity with the aliphatic part of the Thr^207^ side chain. Interestingly, not only NZF tail helix residues but also residues of the core–tail linker such as Tyr^228^ and Pro^230^ participated in interactions with the NZF core, an observation in line with the comparably high backbone stability observed for these linker residues (Fig. 3). In addition, the NZF tail appeared to interact with the core *via* several electrostatic interactions: several negatively charged amino acids of the NZF tail helix were found to directly face positively charged amino acids of the NZF core (Fig. 4*A*). This is highlighted by Glu^234^, the side chain of which points toward a joint surface formed by Lys^205^ and Arg^208^. Likewise, Asp^231^ wraps around the surface formed by Lys^205^. Interestingly, two positively charged residues, Arg^235^ and Arg^237^, do participate in the interaction with the NZF tail, but mostly *via* the aliphatic part of their side chain, not the charged guanidinium groups, which were found to point outward from the structure. This indirect involvement of Arg^235^ in stabilizing the relative arrangement of the NZF core and tail fragments (*i.e.*, not in directly interacting with Ub) also explains the large chemical shift change observed upon M1-linked poly-Ub binding for this residue (Fig. 1*C*) since at this location the helix orientation needs to change when going from a free to an M1-linked poly-Ub-bound state (Fig. 2*D*). While the interactions with the NZF core were numerous in the N-terminal part of the NZF tail helix, virtually no interactions with the NZF tail were found in its C-terminal part as the PISA web server (34) reported zero buried surface area (relative to the NZF core) for residues Ala^239^ to Gln^250^. This observation is in fine agreement with the higher degree of flexibility for the C-terminal part of the helix observed by the conformational dynamics experiments (Fig. 3).

The electrostatic nature of the core–tail interaction was confirmed by calculating the electrostatic potential at the surface of the NZF tail (35) (Fig. 4*B*). The NZF tail contains a total of seven negatively charged amino acids, resulting in a high local negative charge density that is only partially compensated by the presence of three arginine residues (Fig. 4*B*, *left*). As expected from the analysis of the core–tail interface (Fig. 4*A*), positively charged functional groups on the NZF core are positioned to interact with patches of negative charge density on the NZF tail (Fig. 4*B*, *right*). Specifically, the ε-amino group of Lys^205^ and the guanidinium group of Arg^208^ are electrostatically ideally positioned to interact with two patches of negative charge density each. Taken together, electrostatic interactions play a crucial role in stabilizing the relative arrangement of the NZF tail and NZF core.

To further confirm the importance of these electrostatic interactions in stabilizing the HOIL-1L NZF domain, we compared the amino acid sequences of the HOIL-1L NZF domains from various species, ranging from humans and mice to chicken and fish (Fig. 4*C*). The evolutionary conversation of residues important for proximal (indicated in *indigo* in Fig. 4*C*) and distal (indicated in *green* in Fig. 4*C*) Ub binding, as well as Zn^2+^ coordination residues, have already been discussed by Sato *et al.* (16). Interestingly, the residues that engage in electrostatic interactions between the NZF core and the tail in the free form, such as Lys^205^, Arg^208^ (as well as the connecting residues Thr^207^ and Pro^20^^6^), Glu^231^, and Glu^234^, were found to be highly conserved (Fig. 4*C*, *red*), whereas additional residues that introduce negative charge density into the NZF tail, Glu^232^ and Glu^233^, were at least partially conserved among species. In addition, this analysis revealed that the two key residues that specifically orient the NZF core to its tail, Tyr^228^ and Pro^230^, were also highly conserved indicating that a conserved YXP motif in the HOIL-1L NZF, where X provides an outward-facing polar side chain, is crucial for bringing about the correct orientation of NZF tail to the NZF core. Taken together, high conversation in sequence is not only important for preserving the zinc finger structure and the specific (linear) Ub-binding surfaces but also for stabilizing the internal core–linker–tail structural arrangement of the NZF domain itself.

### A conformational switch in the NZF domain

In the visualization of the electrostatic potential of the NZF tail, we made the intriguing observation that the side-chain hydroxyl group of Thr^207^ (Fig. 4*B*), a residue sandwiched between the highly conserved residues Lys^205^, Pro^206^, and Arg^208^ (Fig. 4*C*), came into close proximity with a patch of high negative charge density centered around Glu^234^ of the NZF tail. Not only is Thr^207^ also highly conserved among species but also is the facing residue Glu^234^ as well (Fig. 4*C*). Thr^207^ has been identified as a residue that can undergo phosphorylation in full-length rat HOIL-1L *in vivo* (36) by the action of protein kinase Cβ. Phosphorylation at Thr^207^ reportedly suppressed the catalytic E3 activity of HOIL-1L, which suggests that some critical change in HOIL-1L structure and function must occur upon phosphorylation. In other words, Thr^207^ of HOIL-1L might be a conformational switch, which can be activated by introducing additional negative charge density in the form of a phosphoryl (–PO_3_^2–^) group into the core–tail interface.

**Figure 5:**
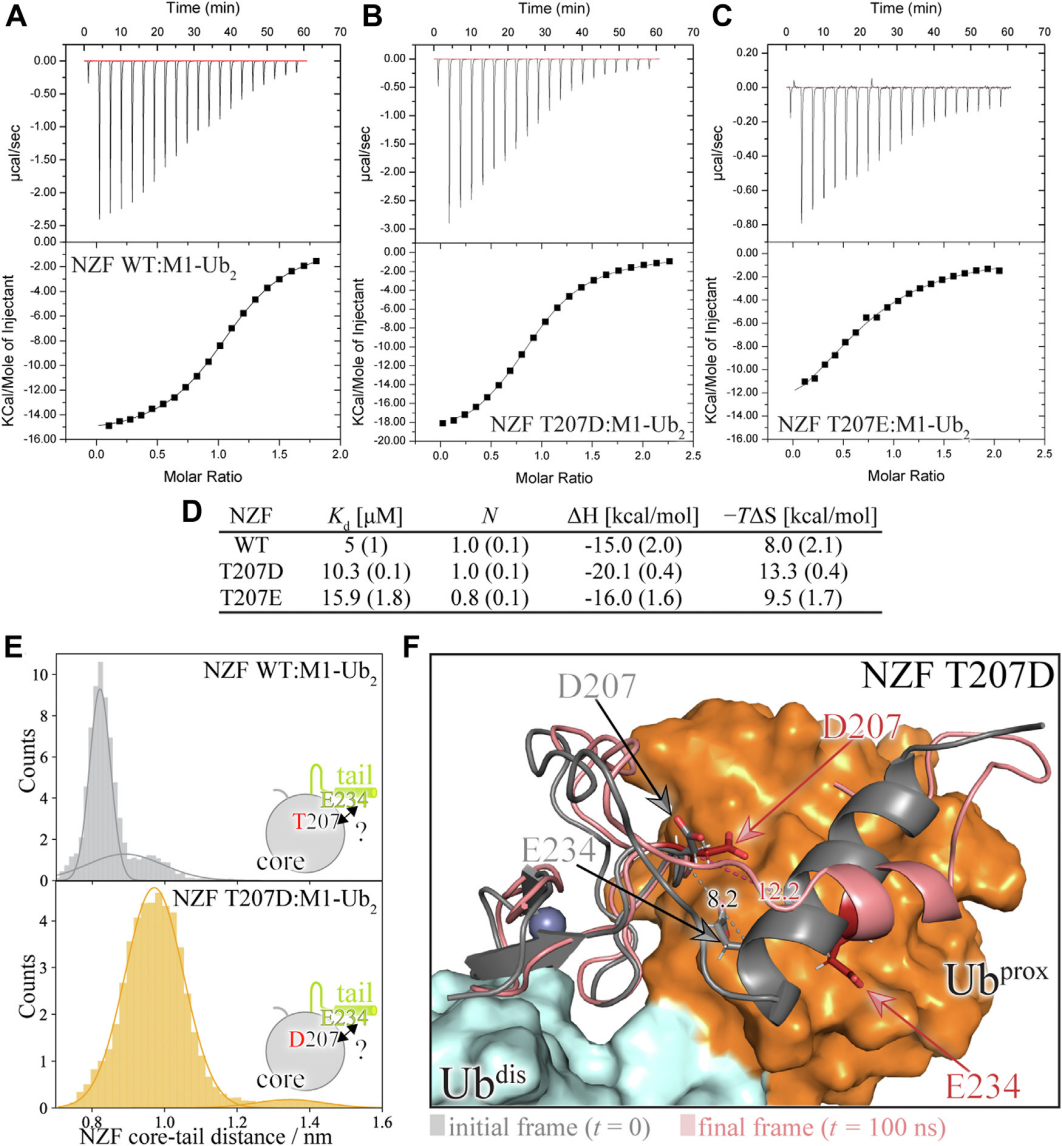
**A conformational switch at the NZF core–tail interface affects polyubiquitin (poly-Ub) binding.** ITC thermograms for (*A*) wildtype, (*B*) T207D, and (*C*) T207E HOIL-1L NZF binding to M1-linked di-Ub are shown. The *upper panels* show the measured raw data (after baseline correction), whereas the *lower panels* display the integrated heat per injection point. *D*, thermodynamic parameters obtained by curve fitting to a one-site exchange model in Origin (see Experimental procedures section). *K*_*d*_, dissociation constant; *N*, stoichiometry; Δ*H*, enthalpy change; Δ*S*, entropy change; and *T*, absolute temperature (298 K). Errors in the derived parameters are given in parenthesis and represent the SEM from three independent experiments. *E*, distribution of distances between the NZF core (measured at Thr^207^/Asp^207^ Cα atom) and the NZF tail (measured at the Glu^234^ Cα atom) in MD simulations for wildtype (*upper panel*) and phosphomimetic (*lower panel*) HOIL-1L NZF domain in the NZF–di-Ub complex. *F*, visualization of the distance variation by two frames taken at the start (*gray*, *t* = 0 ns) and end (*light pink*, *t* = 100 ns) of an example trajectory. The NZF domain is shown in the *cartoon*, whereas M1-linked di-Ub is shown in the *surface representation*. The initial Asp^207^–Glu^234^ distance of 8.2 Å is increased to 12.2 Å with the effect that the carboxylic acid group–containing side chain of Glu^234^ is pushed outward, and unfolding of the C-terminal part of the NZF tail helix is initiated. ITC, isothermal titration calorimetry; MD, molecular dynamics; NZF, Npl4 zinc finger.

To test whether the phosphorylation site discovered in full-length rat HOIL-1L had any measurable effect on the function of the isolated NZF domain that we focus on in this study, we examined the thermodynamics of M1-linked poly-Ub binding of phosphomimetic *versus* wildtype HOIL-1L NZF. Consistent with published SPR data (16) and our NMR titration observations (Fig. 1), wildtype HOIL-1L NZF specifically bound to M1-linked di-Ub (Fig. 5*A*). ITC revealed an exothermic reaction (Fig. 5*D*) with a 1:1 stoichiometry, indicating that one NZF domain binds to one molecule of di-Ub. The dissociation constant was determined to be 5 μM, which agrees well with the observed slow exchange regime. Strikingly, the phosphomimetic mutation T207D decreased the affinity of the NZF domain for M1-linked di-Ub by about twofold (Fig. 5*B*). Although enthalpically binding was even more favorable than in the case of the wildtype NZF domain, a larger entropic penalty counteracted this effect, resulting in a dissociation constant of 10.3 μM (Fig. 5*D*). When Thr^207^ was substituted for Glu instead of Asp, the affinity toward M1-linked di-Ub was further decreased to about threefold (Fig. 5*C*). In addition, the stoichiometry decreased to 0.8 (Fig. 5*D*). This is likely because the longer side chain of Glu^207^ compared with Asp^207^ would bring it closer to Glu^234^, resulting in a larger Coulombic repulsion between these two patches of negative charge density. Such a partial disruption of the integrity of the NZF core–tail interface could also favor dissociation of the NZF–linear di-Ub complex. For instance, the structurally “outward-facing” NZF tail side chain of Glu^233^ contributes to di-Ub binding *via* salt-bridge formation with Lys^63^ of the proximal Ub moiety (Fig. S4), whereas its neighboring residue—Glu^234^, which is “inward facing”—is sensitive to changes in the charge state at residue 207. Taken together, these results show that the phosphorylation site at Thr^207^, originally identified in rat HOIL-1L (36), can indeed form a conformational switch between the NZF core and tail fragments. Its activation by introducing negative charges into the core–tail interface weakens M1-linked di-Ub binding.

To further solidify the link between the integrity of the NZF core–tail interactions, its disruption by an introduced negative charge density, and complexation with M1-linked di-Ub, we conducted a series of 100 ns MD simulations of the NZF–M1-linked di-Ub complex, that is, the bound state. This allowed us to sample multiple independent trajectories with different degrees of distal or proximal Ub motion relative to the NZF and then compare the distribution of the NZF core–tail distances as measured at the Cα atoms of Glu^234^ to the (wildtype) residue Thr^207^ and (mutant) phosphomimetic Asp^207^. As anticipated from the inspection of the electrostatic potential (Fig. 4), the charge density in the NZF core–tail interface also affected the conformational dynamics of the bound state as the distance distribution was strikingly different in wildtype *versus* phosphomimetic HOIL-1L NZF. While wildtype NZF also showed some degree of dynamic motion in the distance distribution of the NZF core to the tail under these conditions, most of the time the core–tail distance was approximately 0.8 nm, closely resembling the short distance in the cocrystal structure (Fig. 5*E*; *upper*). Conversely, for the T207D mutant, the distribution was overall shifted to larger core–tail distances and even some very large distances of >1.2 nm were observed (Fig. 5*E*; *lower*). One example of such a frame is shown in Figure 5*F*, where the charge–charge repulsion appeared to have pushed Glu^234^ out of the initial NZF core–tail interface, destabilizing the helix structure and likely weakening interaction with proximal Ub. Although we do not claim here that these MD simulations perfectly retrace the actual motion occurring in solution, the *relative* difference between wildtype and phosphomimetic NZF under equivalent MD conditions establishes that the integrity of the NZF core–tail interface can be potentially disrupted by phosphorylation, thus also affecting Ub binding.

## Discussion

In this study, we have solved the previously missing solution structure of the free HOIL-1L NZF domain by solution NMR spectroscopy. We have also demonstrated the importance of internal structural dynamics between the NZF core and NZF tail in the specific binding to M1-linked poly-Ub. Interestingly, we found that a previously reported phosphorylation site (Thr^207^) in the HOIL-1L NZF core can act as an important conformational switch that not only disrupts NZF core–tail interactions to displace the helix of the NZF tail but also weakens M1-linked poly-Ub binding affinity.

Surprisingly, the region linking the NZF tail to the NZF core was not completely flexible in solution (Fig. 3). This is likely because of evolutionarily conserved stacking interactions with the NZF core (Fig. 4*A*), and as a result, the NZF tail did not easily detach from the core *via* millisecond dynamics (Fig. 3). Therefore, we can conclude that the core–tail linker is not simply a fully flexible hinge point that would allow the NZF tail to freely sample various orientations in solution to be “on the lookout” for a candidate proximal Ub moiety.

Although in our structural representations, we have retained the helical structural representation for all NZF tail residues as observed in the minimum-energy CYANA structure (Fig. 2), we stress that hnNOE experiments (Fig. 3) and chemical shift data strongly suggested that at least the last five residues of the tail helix are conformationally unstable (secondary structure propensity score <0.4 (31)). This high flexibility in the C-terminal half of the NZF tail helix (Fig. 3) may suggest the presence of a “fly-casting” mechanism (33, 37), in which this part of the helix possesses a higher degree of conformational flexibility than it would have in a rigidly folded helix, which enhances the capture radius for the binding site on a nearby Ub molecule in solution.

**Figure 6:**
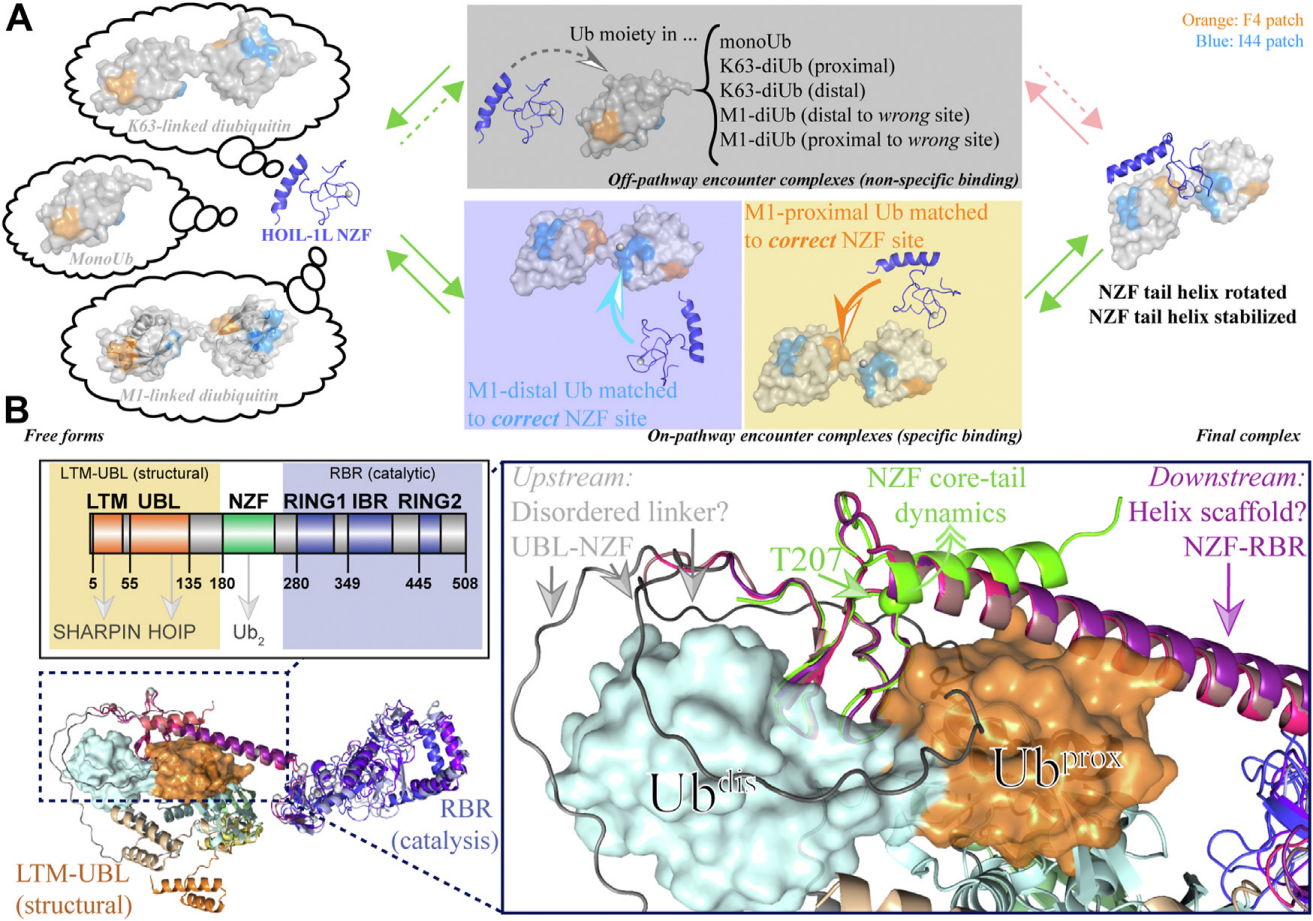
**Interaction of HOIL-1L NZF with ubiquitin (Ub) species may impact global HOIL-1L structure and function.***A*, possible molecular states of HOIL-1L NZF and candidate Ub species before, during, and after binding in solution. *Left*, both (poly)-Ub species (rotatory freedom about its flexible C-terminal tail of distal Ub) and the NZF tail (C-terminal end of helix can be transiently destabilized) have residual dynamics in their free forms, enabling efficient conformational search for candidate binding sites on each other. *Middle*, upon encounter, both correctly matched “on-pathway” Ub-binding sites on the NZF to proximal or distal Ub (*lower panel*) and incorrectly matched “off-pathway” (*upper panel*) encounter complexes can form. Off-pathway complexes also form with Ub species other than the specific target—M1-linked poly-Ub. *Right*, in case of on-pathway binding, the NZF helix is rotated and stabilized, and a specific final complex is formed. *B*, heterogeneous structural predictions for HOIL-1L reveal shared features. Selected AlphaFold predictions for full-length HOIL-1L of human, mouse, and rat are shown. All structures are structurally superimposed (*cartoon representation*) onto the NZF domain and the cocrystal structure of the NZF domain (*green cartoon*) in complex with M1-linked di-Ub (*cyan* and *orange surface representation*) is also shown. Despite the great variability in domain–domain positioning overall, two important common features are the extended length of the NZF tail helix downstream of the NZF domain and the long ∼70-residue unstructured linker upstream of the NZF domain. A crucial role for the NZF in not only poly-Ub binding but also in determining the global arrangement of the catalytic (RBR) domain with respect to the LUBAC-tethering (LTM-UBL) domains is implied. Color code for HOIL-1L domains: RBR: human—*purple*, rat—*blue*, mouse—*light blue*; tethering helix: human—*pink*, rat—*dark magenta*, mouse—*beige*; UBL: human—*cyan*, rat—*light green*, mouse—*light blue*; LTM: human—*yellow*, rat—*orange*, mouse—*light orange*; LTM-NZF linkers: (all species) *gray*. HOIL-1L, heme-oxidized IRP2 ubiquitin ligase 1; NZF, Npl4 zinc finger.

Thus, the exact mechanism of the NZF binding to M1-linked poly-Ub on the level of individual amino acids requires further elucidation, and we plan to dissect the kinetics of the binding mechanism in detail in a future study using the low-ligand concentration (to selectively modulate the *k*_on_[ligand] term of *k*_ex_) relaxation dispersion approach developed by Peter Wright’s group (33, 38) or in-depth titration line shape analysis (39, 40). However, this analysis will be complicated by the need to distinguish cases where the “wrong” Ub is bound to a binding site on HOIL-1L NZF (*e.g.*, where proximal Ub is bound to the distal Ub binding site—*i.e.*, “off-pathway binding”) from “correct” (“on-pathway”) binding (Fig. 6*A*). While this analysis is beyond the scope of the current study, it is likely that the observed weak binding to K63-linked di-Ub and mono-Ub (Table S2) are such off-pathway states that cannot further proceed toward a fully bound state.

To understand the function of the NZF domain in both full-length HOIL-1L and within the context of the ternary LUBAC complex, it will be necessary to obtain structures of full-length HOIL-1L in both the free and HOIP/SHARPIN-bound forms. Although at present, only low-resolution electron microscopy maps (41) and AlphaFold (42) structures are available to hypothesize on a role of the NZF in full-length HOIL-1L from a structural biology perspective, comparing multiple AlphaFold structural predictions for HOIL-1L from several species—thus providing some variance in amino acid sequence—revealed several interesting common features, which might be confirmed by future experimental studies. Although AlphaFold showed a large variation in the relative placement of the upstream (LTM [LUBAC-tethering-motif]-UBL) and downstream (RING1–IBR–RING2) domains as viewed from the perspective of the NZF domain (Fig. 6*B*), it consistently modeled the linker between the UBL and NZF domains as a long unfolded peptide in line with previously conducted domain identification (3, 6, 7, 43), protease cleavage (44), and crosslinking (41) studies on HOIL-1L. The N-terminal LTM and UBL domains are then folded back at various positions onto the remainder of the HOIL-1L structure. In other words, immediately upstream of the NZF core—that is, near the proximal Ub-binding site—proximal Ub binding appears to be possible, if this site is not obstructed by this long linker. Conversely, the distal Ub-binding site (immediately downstream from the NZF domain) might also be bound by other domains of HOIL-1L itself. Although the large variability in the AlphaFold models precludes any definite hypothesis on *which* domain of HOIL-1L could bind and thus occlude the distal Ub-binding site, in the case of another E3 RBR Ub ligase, Parkin, it has been shown that the E3’s own UBL domain (owing to a similar ∼70-residue linker between the UBL and RING0 domains) can bind to the same site on the E3 (45, 46) as Ub and a similar mechanism might exist in HOIL-1L (5, 8).

Another interesting feature of this ensemble of AlphaFold structures is the length of the NZF tail α-helix. While in this study and the previous crystallographic work (16), only a HOIL-1L NZF fragment up until residue Gln^250^ was used, AlphaFold and PSIPRED 4 consistently predict for many species—thus, even in the presence of some variability in sequence—that this helix extends until approximately residue 270 (in human HOIL-1L), thus providing a large central scaffolding helix that connects the N-terminal region (LTM-UBL; involved in both LUBAC tethering (5) and M1-linked poly-Ub binding) to the C-terminal (RBR; involved in catalysis) region. Thus, even though the relative positioning and orientation of these domain fragments cannot be determined from structural predictions alone, these considerations highlight the integral position and importance of the HOIL-1L NZF domain in the context of the yet-unknown full-length structure of HOIL-1L. The conformational switch at Thr^207^ identified here thus likely not only regulates poly-Ub binding (Fig. 5) but also may affect the orientation of the putative long scaffolding helix and thereby the relative positioning of the catalytic (RBR) and tethering (LTM-UBL) domains of HOIL-1L. Without a doubt, future studies will unveil more of the exciting structural complexity of HOIL-1L as a full-length protein, especially in the context of the ternary LUBAC complex.

## Experimental procedures

### Protein expression and purification

N-terminally glutathione-*S*-transferase (GST)-tagged mouse HOIL-1L NZF domain (residues Pro^192^—Gln^250^) encoded on a pGEX-6p1 vector was transformed into *Escherichia coli*–competent cells strain BL21 (*DE3*) using a vector available from previous studies (16, 31). The transformed cells were grown at 37 °C with shaking at 190 rpm in LB medium supplemented with 10 μM zinc sulfate and 50 mg l^−1^ ampicillin as selection pressure. Protein expression was induced in the early midlog phase (absorbance at 600 nm = 0.5) of the growth by the addition of 0.3 mM IPTG (final concentration), and the zinc sulfate concentration was raised to 20 μM. At this point, the temperature was decreased to 15 °C, and the culture was continued for another 24 h before harvesting the culture by centrifugation (5180*g*, 20 min, 4 °C); the resulting pellets were stored at −80 °C.

Pellets were resuspended in lysis buffer consisting of 40 mM HEPES (pH 7.4), 1 mM Tris(2-carboxyethyl)phosphine hydrochloride (TCEP), and 1 μM zinc sulfate, and cell lysis was achieved by ultrasonication. The lysates were subsequently cleared by ultracentrifugation (48,384 rpm, 30 min, 4 °C), and the soluble fraction was applied onto a glutathione sepharose 4 fast flow column (GE Healthcare). The column was washed with four column volumes (CVs) of lysis buffer, 2 CV of lysis buffer with an additional 500 mM sodium chloride, and 2 CV of lysis buffer with an additional 1 M of sodium chloride. At this point, the column was reequilibrated with lysis buffer, and the GST tag was cleaved by incubation with human rhinovirus 3C protease at 4 °C overnight. After proteolytic cleavage between the GST tag and the NZF domain, the NZF domain eluted from the glutathione resin, whereas any residual uncleaved GST-NZF would remain bound to the resin. The column was then washed with another 0.5 CV to elute additional NZF protein. Next, the solution was dialyzed against a buffer consisting of 50 mM Tris–HCl (pH 7.4), 40 mM sodium chloride, and 1 mM TCEP. After dialysis, the solution was applied to a HiTrap Q HP column (GE Healthcare) and subjected to ion exchange chromatography, using a sodium chloride gradient ranging from 40 to 300 mM. Finally, the NZF domain was purified by size-exclusion chromatography using a Superdex 75pg 16/60 column (GE Healthcare) in either gel filtration buffer (10 mM Tris–HCl [pH 8.0], 50 mM sodium chloride, and 5 mM β-mercaptoethanol) or ITC buffer (20 mM HEPES [pH 7.0], 50 mM sodium chloride, and 1 mM TCEP). The final purity as assessed by SDS-PAGE Coomassie brilliant blue staining was 99%, and MALDI-TOF mass spectrometry confirmed the expected molecular weight.

To produce the NMR samples with uniform ^13^C and ^15^N labeling, the HOIL-1L NZF culture was carried out under equivalent conditions using M9 minimal media harboring [*U*-^13^C] glucose and [^15^N] ammonium chloride (Cambridge Isotope Laboratories) as the sole carbon and nitrogen sources, respectively. Point mutation–carrying plasmids of the NZF domain (T207D and T207E) were created by polymerase chain reaction, and the mutant NZF proteins were expressed using autoinduction in N-5052 media at 20 °C for 60 h (8, 47). K63- and M1-linked di-Ub were prepared as previously described (48).

### General NMR spectroscopy

NMR measurements were carried out on a Bruker Avance II spectrometer (^1^H Larmor frequency: 700 MHz) equipped with a 5 mm ^15^N/^13^C/^1^H *z*-gradient triple resonance cryoprobe (Bruker BioSpin). Before NMR measurements, the NZF domain was buffer exchanged into NMR buffer (20 mM HEPES [pH 7.0], 50 mM sodium chloride, 1 mM TCEP, and 5% deuterium oxide) and placed in Shigemi tubes at a final sample volume of 300 μl. The concentration of the NZF domain for triple resonance experiments was 1 mM, whereas the starting concentration for the titration experiments was 0.1 mM. The details of the concentrations during the titration experiments are indicated in the figure legends. While we had reported the details of the triple resonance experiments employed for assignment of the NZF domain in its free and M1-linked di-Ub-bound forms before (31), here we performed additional NOESY experiments (mixing time: 150 ms) to guide the structure calculation: ^15^N-edited NOESY–HSQC (heteronuclear single quantum coherence) and ^13^C-edited NOESY–HSQC (49). The ^1^H chemical shift was calibrated using 2,2-dimethyl-2-silapentane-5-sulfonate, and the heteronuclear ^13^C and ^15^N shifts were calibrated indirectly with respect to the proton chemical shift (50). Data acquisition was done using Bruker TopSpin (Bruker), processing of the NMR data was done using NMRPipe (51), and spectrum analysis was conducted in CcpNmr Analysis, version 2.4.1 (52).

In the hnNOE experiments (Bruker pulse program *hsqcnoef3gpsi3d*), the recycle delay was set to 5 s (∼10 *T*_1_ (^15^N)) to assure sufficient spin-lattice relaxation and thus restoration of equilibrium magnetization between acquisitions. Saturation of ^1^H was performed by a series of 120° ^1^H pulses during the recycle delay, that is, over a time of 5 s; saturation was turned off in the control experiment. The free NZF domain was measured at a concentration of 1.0 mM. The sample concentrations for measuring hnNOE values in the M1-linked di-Ub-bound form of the NZF were 0.5 mM NZF and 2 mM di-Ub, respectively. Two independent measurements were carried out to derive average values and the standard error of the mean. ^15^N relaxation dispersion measurements used the pulse sequence *hsqcNr2rex3d* employing a Carr–Purcell–Meiboom–Gill relaxation time of 50 ms with τ_CP_ values of 0, 25, 12.5, 8.33, 6.25, 4.17, 3.12, 2.08, 1.79, 1.56, 1.39, 1.04, 0.78, 0.61, and 0.50 ms. The pulse sequence is available under the URL http://www.moleng.kyoto-u.ac.jp/∼moleng_01/nmr/r2.html or upon request to the authors. Data points at τ_CP_ values of 0, 8.33, 1.04, and 0.50 ms were measured twice as a base for calculating experimental uncertainties. Transverse ^15^N relaxation measurements were conducted as previously described (53, 54) by using the Carr–Purcell–Meiboom–Gill pulse train. Delays for the *R*_2_ experiments were 0, 0.016, 0.032, 0.048, 0.064, 0.08, 0.112, and 0.144 s. Data points at 0, 0.048, and 0.096 s were measured twice as a base for calculating experimental uncertainties. The GLOVE (55) package was used for least-squares estimation of *R*_ex_ and *R*_2_ and their uncertainties (from Monte–Carlo simulations with 100 iterations).

### NMR titration experiments

In the analysis of the NMR titration experiments, the amide CSP was calculated in the form of an averaged value of the ^1^H and ^15^N chemical shift changes as (25):

$$CSP=\sqrt{{{\Delta \delta }_{H}}^{2}+{\left(0.1{\Delta \delta }_{N}\right)}^{2}}$$

where ${\Delta \delta }_{H}$ and ${\Delta \delta }_{N}$ represent the individual chemical shift changes in the proton and nitrogen dimensions, respectively, and the factor 0.1 reflects the difference in chemical shift dispersion between ^1^H and ^15^N.

### Solution structure determination

CYANA, version 3.98.13, was used for NMR structure calculation (56, 57, 58). Input peak lists were obtained by peak picking the ^13^C- and ^15^N-edited 3D NOESY–HSQC spectral strips in CcpNmr Analysis from root resonances in the respective HSQC spectra. An initial NOE- and dihedral angle (φ/ψ angles obtained from TALOS+ (59)) restraint-based structure was obtained in CYANA, and this structure was then further refined in CYANA against a set of RDCs.

To obtain the dataset of RDC constants, a PEG bicelle alignment medium was prepared by mixing 50 μl of pentaethylene glycol monododecyl ether (C_12_E_5_ PEG; Sigma–Aldrich) with 200 μl of NMR buffer (20 mM HEPES [pH 7.0], 50 mM sodium chloride, and 1 mM TCEP) and 50 μl D_2_O and then stepwise adding small aliquots (1 μl) of hexanol (Sigma–Aldrich) with vigorous vortexing after each addition. The final amount of hexanol added was approximately 16 μl, at which point a clear solution was obtained. The PEG bicelle solution was then mixed 1:1 with protein solution, and the final solution was allowed to equilibrate in the NMR magnet for at least 1 h before measurements.

Two independent in-phase anti-phase-HSQC (60) experiments each in the presence and absence of the nematic phase were used to estimate the RDC (*D*_NH_) and scalar coupling (*J*_NH_) values. Average values of the obtained *D*_NH_ values were used in the subsequent analysis, and the uncertainties in *D*_NH_ were estimated as the SEM among the two measurements.

The alignment tensor was estimated using the initial CYANA structure (see aforementioned) independently in the program REDCAT (61) and CYANA using the program-provided macro *FindTensor.cya*. For this determination, only a small number of RDCs was chosen, and the correlation coefficient for this initial tensor estimate was 0.99. For structure determination, the number of RDC restraints used was gradually increased (see the supporting information for final structure calculation restraints and statistics). The final structure calculation protocol was executed independently 200 times with different random number seeds in the CYANA protocol to verify the integrity of the obtained result. Structural representations were drawn in PyMOL (Schrödinger, LLC), and protein BLAST sequence alignments were visualized using BoxShade (https://github.com/pinbo/boxshade).

### ITC

The binding between linear di-Ub and the HOIL-1L NZF domain (wildtype or point mutant) was studied by ITC experiments at 298 K on a MicroCal iTC200 system (GE Healthcare). Both ligand and analyte proteins were dialyzed against ITC buffer overnight at 277 K and thoroughly degassed before each experiment. For most experiments, the concentration of linear di-Ub in the syringe was 1 mM, and the sample cell contained 100 μM of HOIL-1L NZF, whereas several experiments used half of these concentrations. A total of 19 injections of 2 μl each was carried out at 3 min intervals, and an initial 0.4 μl injection was discarded. The measured thermograms were processed using Origin 7 (OriginLab Corporation). Three independent experiments were conducted each, and the experimental data were fitted to a binary (“one set of sites”) binding model in Origin.

### MD

MD simulations were conducted in GROMACS, version 2021 (62) under the amber99sb-ildn (63) force field at 300 K as described before (8, 53, 64). The initial structure was obtained from the crystal structure of the HOIL-1L NZF domain in the M1-linked di-Ub-bound form (PPDB ID: 3B08 (16)). Simulations were conducted in TIP4P/2005 water at a sodium chloride concentration of 50 mM. Distance restraints between the Zn^2+^ ion and the coordinating cysteine Sγ atoms (*r*_1_ = 2.3 Å; r_2_ = 3.0 Å) were applied. To achieve adequate sampling of the conformational space, 20 independent 100 ns simulations were conducted, and analysis of trajectories was carried out using tools included in the GROMACS package.

## Data availability

Atomic coordinates have been deposited with the RCSB PDB with the PDB ID code of 8IM5. All data are contained within this article and available from the corresponding authors on reasonable request.

## Supporting information

This article contains supporting information (16, 20, 21, 24, 31, 55, 63, 65, 66, 67, 68).

## Conflict of interest

The authors declare that they have no conflicts of interest with the contents of this article.
