# Supplementary material for: Solution structure of the HOIL-1L NZF domain reveals a conformational switch regulating linear ubiquitin affinity
Source: J Biol Chem. 2023 Aug 16;299(9):105165. doi: 10.1016/j.jbc.2023.105165 (PMC10511788; doi:10.1016/j.jbc.2023.105165)
Supplement: Supporting Tables S1–S5 and Figures S1–S4 [file mmc1.docx]

**Supplementary Information**

**Supplementary Table S1. Comparison of the radius of gyration (*R*_g_) of diubiquitin obtained from MD simulations (this study) and published SAXS data.**

|  | *R*_g_ (Å) | |
| --- | --- | --- |
| diubiquitin | SAXS | MD |
| K63^#^ | 19.0 ± 0.8^a^ | 18.3 ± 0.2 |
| M1 | 20.2 ± 1.3^b^ | 19.0 ± 0.2 |
| ^a^ reference (1), ^b^ reference (2) | | |

^#^ In addition, Liu et al. (3) also find K63-linked diubiquitin to be much more compact than would be expected from the often-cited extended conformation (PDB ID 2JF5). Closed conformers were also described by Ye et al. (4).

**Supplementary Table S2. Affinity of the HOIL-1L NZF domain for K63-linked diubiquitin and monoubiquitin**

|  | *K*_d_ (μM) | |
| --- | --- | --- |
| Ubiquitin type | NMR  (this study) | SPR^c^  (from reference (5)) |
| monoUb | 131 ± 7^a^ | 462 ± 19 |
| K63-linked diubiquitin | 224 ± 8^b^ | 330 ± 13 |
| ^a^, Uncertainties in the derived *K*_d_ values were estimated by the jackknife method in GLOVE.  ^b^, Approximate value obtained from subjecting the titration data series shown in Fig. 1 of the main text to fitting to a 1-site binding fast-exchange model (function TITR_classic2) in GLOVE (6) (7). The stoichiometry parameter bmol was fixed to 1 (i.e., 1 NZF molecule binding to 1 molecule of K63-linked diubiquitin) and the *K*_d_ was defined as a global parameter. While this model fits well for monoUb binding to the HOIL-1L NZF domain, for several residues, it did not satisfactorily describe the observed chemical shift changes for K63-linked diubiquitin. This was due to (*a*) those titration data points showing a slightly sigmoidal shape (i.e., smaller chemical shift changes at low molar equivalents of diubiquitin added), and (*b*) the chemical shift changes not converging for several residues at the end of the titration (at 1.5 molar equivalents added) due to the weak affinity (varying bmol not changing the results). A more complex process, involving high populations of closed conformers of K63-linked diubiquitin (3, 4), appears to take place and contrary to the case of monoUb, and a more sophisticated model based on the Bloch-McConnell (8) equations is necessary to describe this; that is beyond of the scope of this analysis, which aimed only at demonstrating the overall weakness of the affinity and does not cover the detailed complexities of the interaction.  ^c^ In the SPR analysis, GST-NZF was used instead of the isolated (i.e., tag-free) NZF domain. | | |

**Supplementary Table S3. Structure calculation of the HOIL-1L NZF domain**

| **Distance restraints**  Total  Sequential [\|*i - j*\|] = 1  Medium range [\|*i - j*\|] ≤ 4  Long range [\|*i - j*\|] > 4 | 1430  649  344  437 |
| --- | --- |
| **Angular restraints**  Residual dipolar coupling restraints | 28 |
| **Statistics for structure ensemble calculation**  Backbone atoms^a^ r.m.s.d.  All heavy atoms^a^ r.m.s.d.  Number of dihedral angle violations  Number of distance violations (> 0.25 Å) | 0.42 ± 0.12 Å  0.81 ± 0.09 Å  None  None |
| Number of van-der-Waals violations (> 0.3 Å) | None |
| Number of RDC violations (> 0.3 Hz) | None |
| **Ramachandran plot statistics (%)**  Residues in most favored regions  Residues in additional allowed regions  Residues in generously allowed regions  Residues in disallowed regions^b^ | 64.7 %  32.0 %  3.0 %  0.3 % |

^a^Excluding plasmid-linker derived residues, as well as the 3 carboxy-terminal residues.

^b^The single outlier was one plasmid-derived serine residue for which no restraints were available, i.e., not a residue of the HOIL-1L NZF domain.

**Supplementary Table S4. ^15^N-^1^H Residual dipolar couplings of the NZF domain measured in PEG bicelles.**

| Residue | NZF element | DSSP^#^ | D_HN_ / Hz (PEG bicelles)* |
| --- | --- | --- | --- |
| V193 | core |  | 0.15 (0.25) |
| G194 | core | S | 0.46 (0.18) |
| W195 | core | E | -5.00 (0.18) |
| Q196 | core | E | 4.22 (0.46) |
| C197 | core |  | -4.51 (0.39) |
| P198 | core | T | - |
| G199 | core | T | -2.38 (0.11) |
| C200 | core | T | 5.11 (0.18) |
| T201 | core |  | -2.52 (0.53) |
| F202 | core |  | 0.28 (0.92) |
| I203 | core | E | -1.77 (0.07) |
| N204 | core | E | -5.43 (0.18) |
| K205 | core |  | 6.17 (0.14) |
| P206 | core | T | - |
| T207 | core | T | -4.33 (0.57) |
| R208 | core |  | -7.91 (0.75) |
| P209 | core | S | - |
| G210 | core | B | -6.14 (0.32) |
| C211 | core |  | 6.24 (0.28) |
| E212 | core | T | 3.51 (0.11) |
| M213 | core | T | 2.84 (0.57) |
| C214 | core | T | 9.83 (1.24) |
| C215 | core |  | -2.91 (0.15) |
| R216 | core |  | -2.24 (0.32) |
| A217 | core | B | -2.59 (0.25) |
| R218 | core |  | -7.17 (0.35) |
| P219 | core |  | - |
| E220 | core | T | 4.86 (0.11) |
| T221 | core | T | 10.25 (0.53) |
| Y222 | core |  | 2.34 (0.18) |
| Q223 | core |  | -7.56 (0.32) |
| I224 | linker |  | -4.36 (0.18) |
| P225 | linker |  | - |
| A226 | linker | T | -4.54 (0.21) |
| S227 | linker | T | -4.90 (0.35) |
| Y228 | linker |  | -3.76 (0.07) |
| Q229 | linker |  | -4.47 (0.28) |
| P230 | linker |  | - |
| D231 | tail |  | 8.12 (1.53) |
| E232 | tail | H | -5.57 (0.46) |
| E233 | tail | H | -7.42 (0.11) |
| E234 | tail | H | -8.61 (0.67) |
| R235 | tail | H | -6.32 (0.35) |
| A236 | tail | H | -5.71 (0.96) |
| R237 | tail | H | -4.90 (1.06) |
| L238 | tail | H | -5.25 (0.99) |
| A239 | tail | H | -6.10 (0.35) |
| G240 | tail | H | -9.01 (0.14) |
| E241 | tail | H | -6.63 (0.39) |
| E242 | tail | H | -4.90 (0.28) |
| E243 | tail | H | -3.02 (0.46) |
| A244 | tail | H | -6.24 (1.14) |
| L245 | tail | H | -4.65 (0.32) |
| R246 | tail | H | -5.18 (0.10) |
| Q247 | tail | H | -2.59 (0.40) |
| Y248 | tail |  | -3.12 (0.14) |
| Q249 | tail |  | 0.50 (0.07) |
| Q250 | tail |  | 0.78 (0.35) |
| Alignment Tensor |  |  | Value / Hz |
| Magnitude |  |  | 5.31 (0.15) |
| Rhombicity |  |  | 0.17 (0.02) |

*, Values in parentheses indicate the standard error of the mean.

^#^, secondary structure as assigned by the DSSP program (9) given the published crystal structure (PDB ID 3B08) (5). Letters denote: H = α-helix; E = β-strand; B = isolated β-bridge; T = (hydrogen-bonded) turn; S = bend.

**Supplementary Table S5. Comparison of dihedral angles for the core-tail linker region between free and M1-linked diubiquitin-bound structures of HOIL-1L NZF**

| Residue | NZF element | Φ (complex)  / degrees | Φ (free)  / degrees | ΔΦ  / degrees |
| --- | --- | --- | --- | --- |
| Q233 | core | -115 | -125 | 10 |
| I224 | linker | -60 | -95 | 35 |
| P225 | linker | -58 | -70 | 12 |
| A226 | linker | -76 | -64 | 12 |
| **S227** | linker | -146 | -91 | **55** |
| **Y228** | linker | -54 | -56 | **2** |
| **Q229** | linker | -119 | 65 | **184** |
| P230 | linker | -68 | -70 | 2 |
| D231 | tail | -61 | -53 | 8 |
| Residue | NZF element | Φ (complex)  / degrees | Φ (free)  / degrees | ΔΦ  / degrees |
| Q233 | core | 139 | 159 | 19 |
| I224 | linker | 143 | 132 | 12 |
| P225 | linker | 161 | 138 | 24 |
| A226 | linker | -4 | -33 | 29 |
| **S227** | linker | 13 | 24 | **11** |
| **Y228** | linker | 122 | -64 | **186** |
| **Q229** | linker | 98 | 82 | **17** |
| P230 | linker | 152 | 140 | 12 |
| D231 | tail | 165 | 172 | 8 |

Comparative analysis has been performed using the co-crystal structure (ref. (5); PDB ID 3B08) and the free NMR structure reported in this study (lowest-energy conformer). Dihedrals have been obtained in PyMOL (Schrödinger, LLC.) using the *phi_psi* function yielding Φ and ψ for the free and bound forms. The differences between the free and bound forms in Φ and ψ and is shown in the columns ΔΦ and Δψ, respectively. Purple highlighting: the largest difference in linker dihedrals is found in the sequence Ser^227^-Tyr^228^-Gln^229^, which is in fine agreement with the chemical shift perturbation for these residues observed in the titration experiments (Fig. 1 of the main text; Supplementary Fig. S3).

**Supplementary Figures**

**
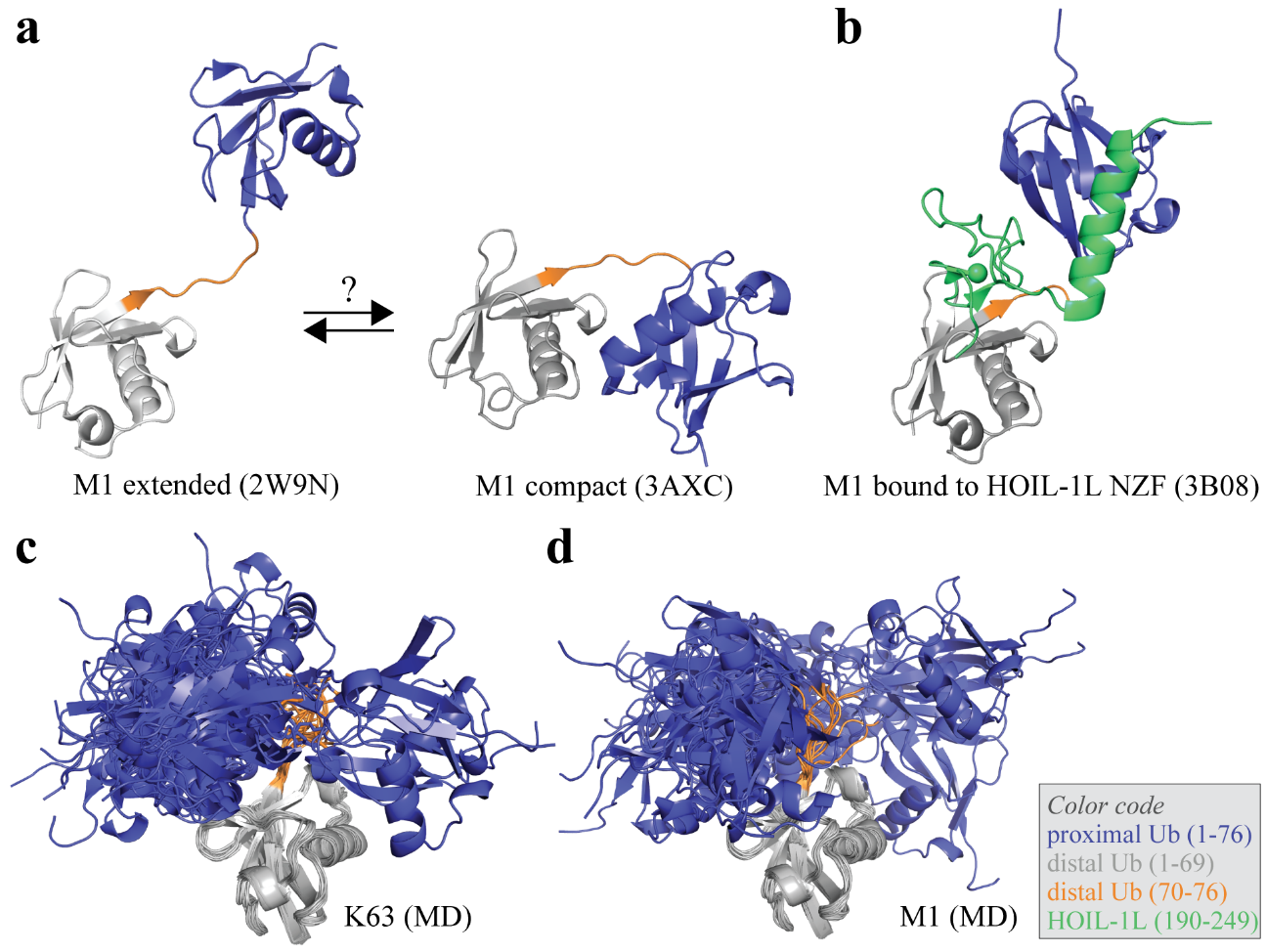
**

**Supplementary Figure S1. Understanding how proteins choose between two dynamic ubiquitin chains.** There are different conformations (open and closed) in two particular types of ubiquitin chains: M1-linked and K63-linked. (**a**) Several open and closed conformers have been described for M1-linked diubiquitin. The most often cited extended crystal structure (PDB ID 2W9N) and a less-frequently discussed compact conformation (PDB ID 3AXC) are shown. Very similar extended and compact structures have been described for the K63-linked chain. Since solution-state experiments have reported a rather broad distribution of conformations (4), an equilibrium between closed and open conformations ought to exist. This begs the question of how ubiquitin-binding domains discriminate such dynamic ensembles when selecting a particular type of ubiquitin chain. This study focuses on the NZF domain of HOIL-1L (**b**), which reportedly binds to M1-linked ubiquitin chains but rejects K63-linked chains (5). Even though both types of chains are dynamic and switch between open and closed conformations, this protein can pick one type while ignoring the other. In (**c**) and (**d**) the dynamics of the two types of diubiquitin are visualized. Shown are snapshots from 1-μs MD simulations of linked diubiquitin under the amber99sb-ildn forcefield (10). From six independent simulations, snapshots of K63-linked (**c**), and M1-linked (**d**) diubiquitin at 300 ns, 600 ns, and 900 ns are shown with the distal diUb subunit superimposed. Orange indicates the flexible carboxyterminal tail of the distal ubiquitin subunit; grey indicates distal ubiquitin; blue indicates proximal ubiquitin. A large degree of domain-domain conformational flexibility owing to the flexible carboxyterminal tail of the distal subunit is evident. We briefly note that other computational studies with similar conclusions are referenced in the main text.


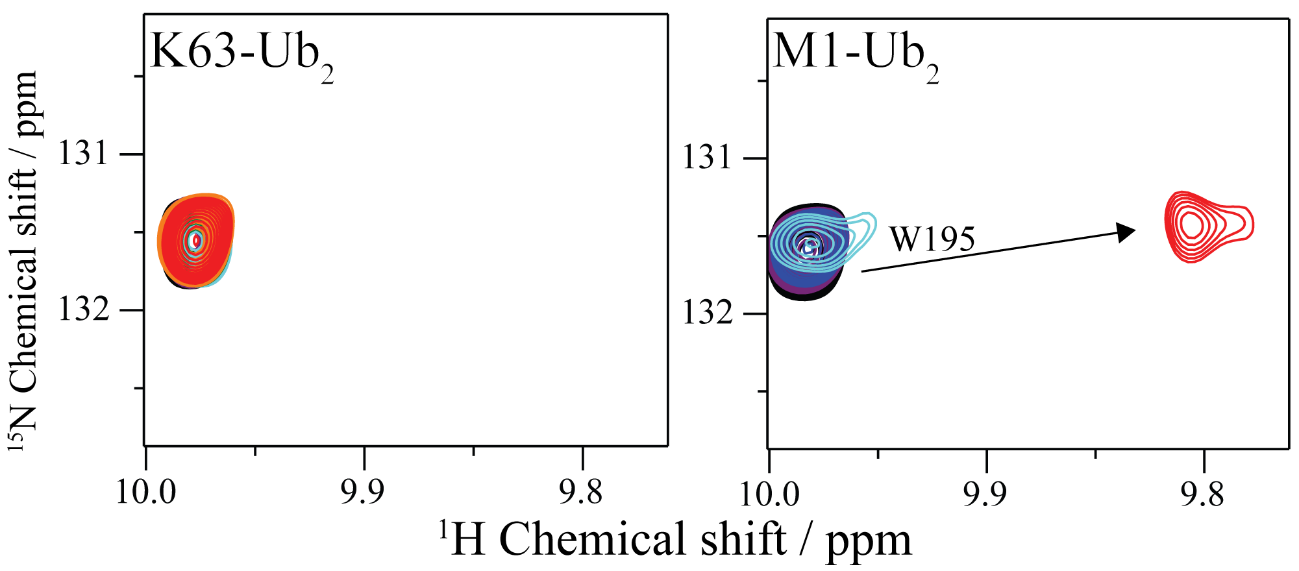


**Supplementary Figure S2. Differential binding of the HOIL-1L NZF domain to M1- and K63-linked diubiquitin.** In addition to the main chain residues shown in Fig. 1 of the main text, here the indole NH resonance of Trp^195^ is shown. As in Fig. 1 of the main text, the titration ranged from 0 to 1.5 eq. of diubiquitin. Protein concentrations (in μM) of HOIL-1L NZF to (:) diubiquitin are 100:0 (0 eq.), 99.3:9.9 (0.1 eq.), 98.7:19.7 (0.2 eq.), 97.4:39.0 (0.4 eq.), 94.9:75.9 (0.8 eq.), 93.8:93.8 (1.0 eq.), 90.9:136.4 (1.5 eq.)].


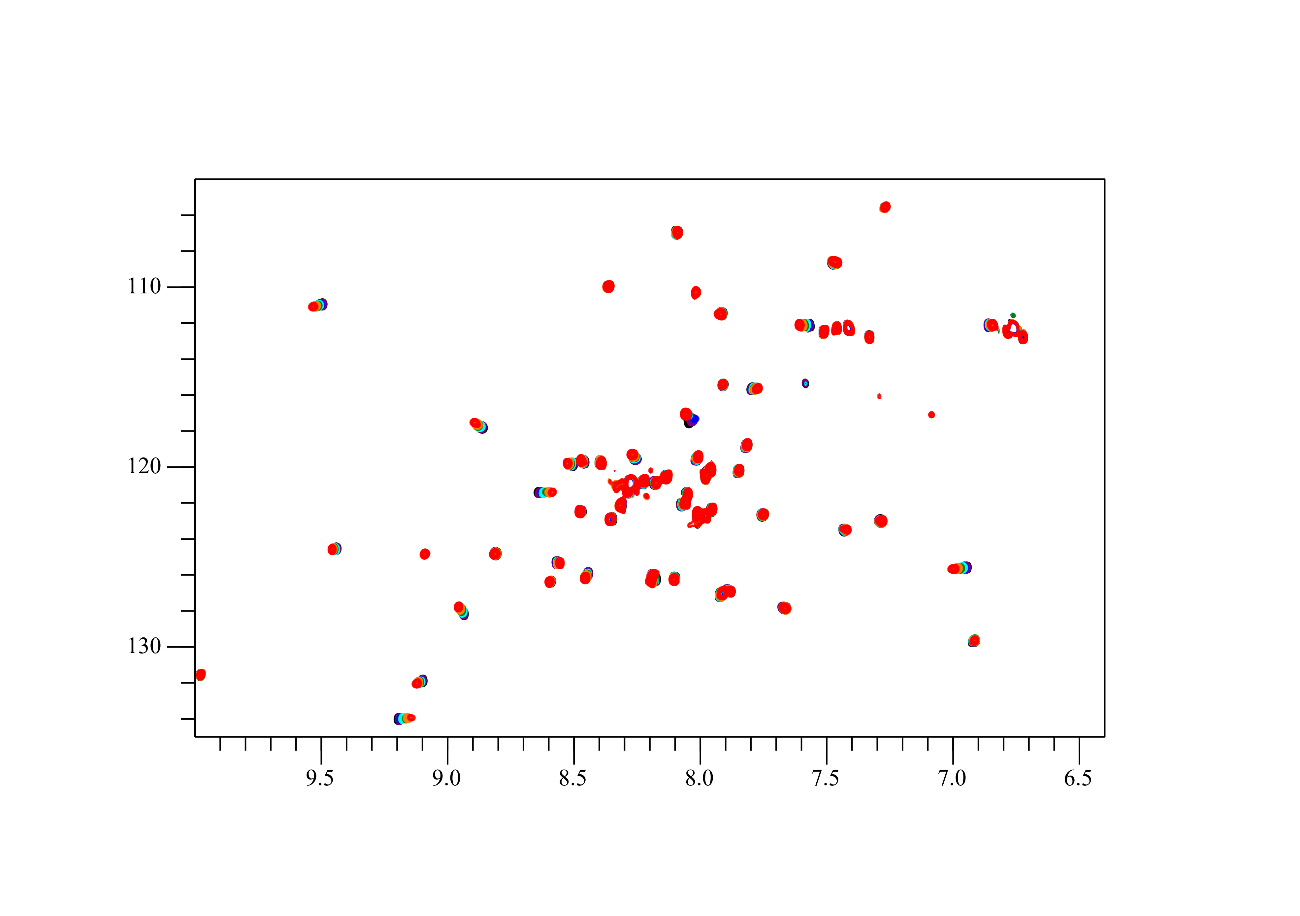

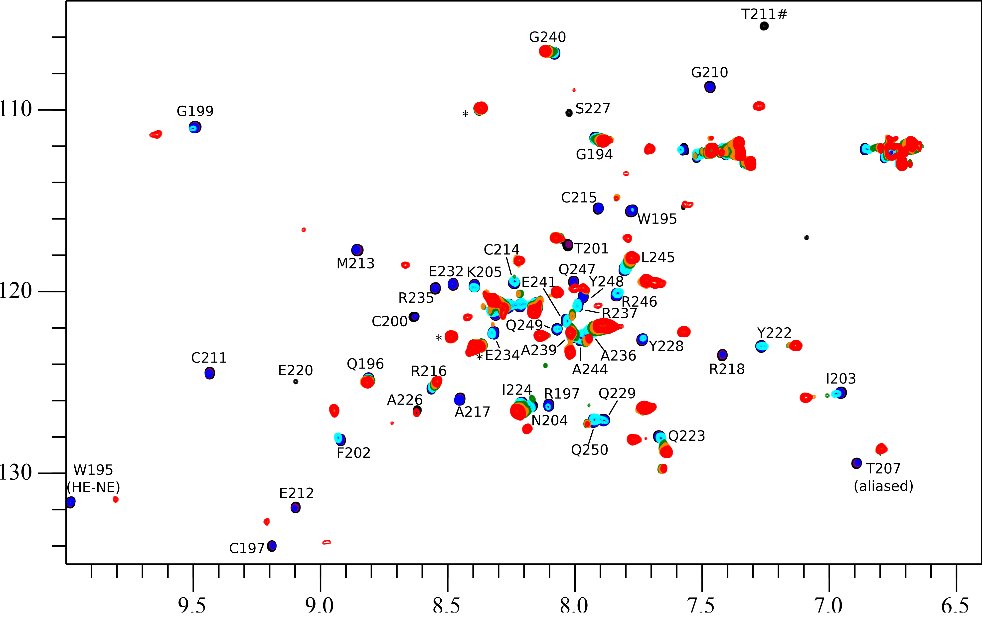


^15^N Chemical shift / ppm

^1^H Chemical shift / ppm

**Supplementary Figure S3.** Overall views of the titration series HSQC spectra depicted in Fig. 1 and Supplementary Fig. S2 are shown for the titration of the HOIL-1L NZF with K63-linked diubiquitin (**upper panel**); the titration of the HOIL-1L NZF with M1-linked diubiquitin (**lower panel**). Colors and protein concentrations are identical to those given in Fig. 1 of the main text and Supplementary Figure S2. While all peaks can be easily traced in the fast-exchange case of K63-linked diubiquitin, many peaks show large slow-exchange displacements that are not easily traced by the titration experiment alone in the case of M1-linked diubiquitin. For this reason, we had previously separately assigned the free and M1-linked diubiquitin bound states of the HOIL-1L NZF domain using triple resonance experiments; chemical shift assignments for the free and bound states from separate triple resonance NMR assignments using a higher concentration of sample are provided in (11) (BMRB entries 27606 and 12026). In the lower panel, cross-peaks are labeled according to the chemical shift in the free form. Peaks labeled with an asterisk (*) are plasmid-derived amino acids. The # denotes that Thr^221^ is clearly observed in the 1.5 eq. condition (red spectrum), but due to line broadening the peak is not visible at the contour levels shown here. Residues in the crowded center of the spectrum that were omitted from labeling due to heavy overlap [these peaks are depicted in a labeled form in (11)] are (from left to right; i.e., from high to low ^1^H chemical shift): Leu^238^, Glu^233^, Glu^243^, Glu^242^, Val^193^, and Asp^231^.


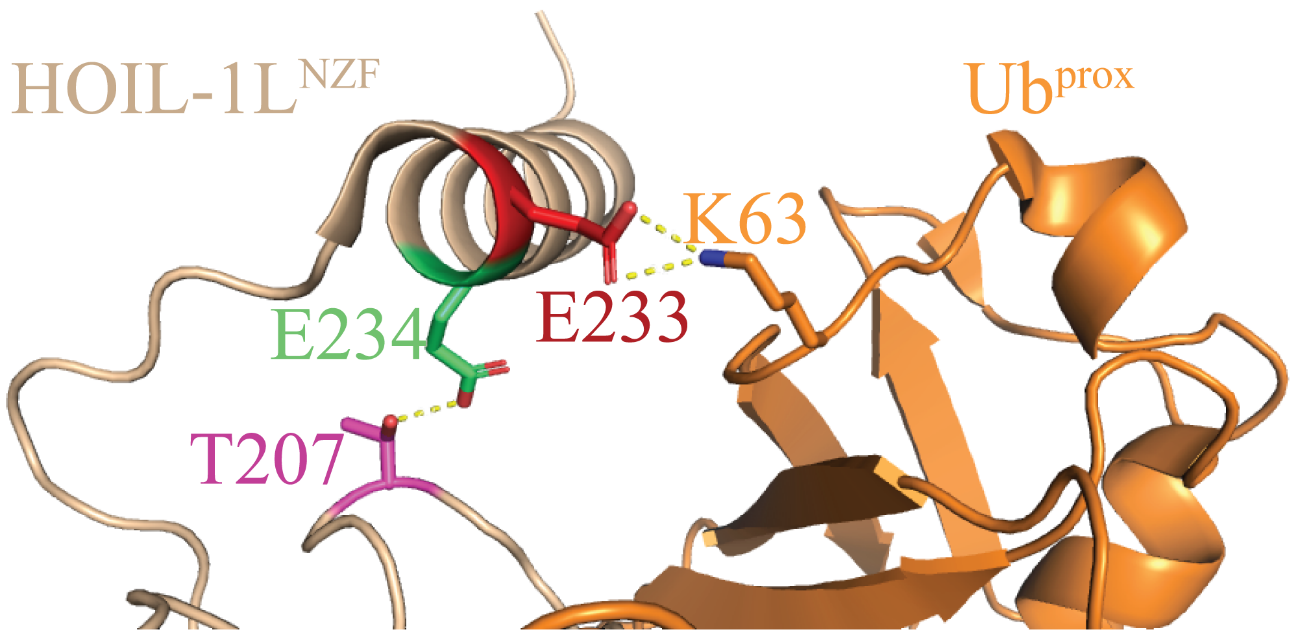


**Supplementary Figure S4. Coupling of NZF core-tail structural integrity and linear diubiquitin binding via electrostatic interactions.** The NZF domain is shown in teal with the proximal moiety of bound linear diubiquitin in orange (structure drawn from the co-crystal structure PDB ID 3B08). The sidechain of Thr^207^ (pink) of the NZF core is in close proximity (sidechain O-O distance 2.7 Å) to that of Glu^234^ (light green) of the NZF tail. Additionally, the adjacent residue Glu^233^ forms a salt bridge with Lys^63^ (orange; sidechain O-N distance of 3.1 Å) of the proximal ubiquitin moiety of linear diubiquitin. The juxtaposition of Glu^233^ and Glu^234^ suggests that charge modulation at Thr^207^ may not only affect NZF core-tail structural integrity, but also negatively impact linear diubiquitin binding either directly by charge-charge repulsion or indirectly by altering the orientation of the tail helix.

**Supplementary References**

1. Tenno, T., Fujiwara, K., Tochio, H., Iwai, K., Morita, E. H., Hayashi, H., Murata, S., Hiroaki, H., Sato, M., Tanaka, K., and Shirakawa, M. (2004) Structural basis for distinct roles of Lys63- and Lys48-linked polyubiquitin chains. *Genes Cells*. **9**, 865–875

2. Thach, T. T., Shin, D., Han, S., and Lee, S. (2016) New conformations of linear polyubiquitin chains from crystallographic and solution-scattering studies expand the conformational space of polyubiquitin. *Acta Crystallogr Sect D Biol Crystallogr*. **72**, 524–535

3. Liu, Z., Gong, Z., Cao, Y., Ding, Y. H., Dong, M. Q., Lu, Y. B., Zhang, W. P., and Tang, C. (2018) Characterizing Protein Dynamics with Integrative Use of Bulk and Single-Molecule Techniques. *Biochemistry*. **57**, 305–313

4. Ye, Y., Blaser, G., Horrocks, M. H., Ruedas-Rama, M. J., Ibrahim, S., Zhukov, A. A., Orte, A., Klenerman, D., Jackson, S. E., and Komander, D. (2012) Ubiquitin chain conformation regulates recognition and activity of interacting proteins. *Nature*. **492**, 266–270

5. Sato, Y., Fujita, H., Yoshikawa, A., Yamashita, M., Yamagata, A., Kaiser, S. E., Iwai, K., and Fukai, S. (2011) Specific recognition of linear ubiquitin chains by the Npl4 zinc finger (NZF) domain of the HOIL-1L subunit of the linear ubiquitin chain assembly complex. *Proc Natl Acad Sci U S A*. **108**, 20520–20525

6. Sugase, K., Konuma, T., Lansing, J. C., and Wright, P. E. (2013) Fast and Accurate Fitting of Relaxation Dispersion Data Using the Flexible Software Package GLOVE. *J Biomol NMR*. **56**, 275–283

7. Walinda, E., Morimoto, D., Sugase, K., Konuma, T., Tochio, H., and Shirakawa, M. (2014) Solution structure of the ubiquitin-associated (UBA) domain of human autophagy receptor NBR1 and its interaction with ubiquitin and polyubiquitin. *J Biol Chem*. **289**, 13890–13902

8. McConnell, H. M. (1958) Reaction Rates by Nuclear Magnetic Resonance. *J Chem Phys*. **28**, 430–431

9. Kabsch, W., and Sander, C. (1983) Dictionary of Protein Secondary Strucutre: Pattern Recognition of Hydrogen-Bonded and Geometrical Features. *Biopolymers*. **22**, 2577–637

10. Lindorff-Larsen, K., Piana, S., Palmo, K., Maragakis, P., Klepeis, J. L., Dror, R. O., and Shaw, D. E. (2010) Improved side-chain torsion potentials for the amber ff99SB protein force field. *Proteins*. **78**, 1950–1958

11. Ishii, N., Walinda, E., Iwakawa, N., Morimoto, D., Iwai, K., and Sugase, K. (2018) NMR resonance assignments of the NZF domain of mouse HOIL-1L free and bound to linear di-ubiquitin. *Biomol NMR Assign.* **13***,* 149–153
